# Macrovascular Endothelial Cells Enhance the Motility of Liver Cancer Cells by Up-regulation of MMP-3, Activation of Integrin/FAK Signaling Pathway and Induction of Non-classical Epithelial-mesenchymal Transition

**DOI:** 10.7150/jca.38209

**Published:** 2020-02-03

**Authors:** Song-Ming Ding, Ai-Li Lu, Jian-Fang Lu, Xu-Liang Chen, Muhammad Ibrahim Alhadi Edoo, Lin Zhou, Hai-Yang Xie, Shu-Sen Zheng, Qi-Yong Li

**Affiliations:** 1 Division of Hepatobiliary and Pancreatic Surgery, Shulan (Hangzhou) Hospital, Hangzhou, Zhejiang, P.R. China; 2 Division of oncology department, First Affiliated Hospital, Zhejiang University School of Medicine, Hangzhou, Zhejiang, P.R. China; 3 Key Laboratory of Combined Multi-organ Transplantation, Ministry of Public Health; Key Laboratory of Organ Trans-plantation, Zhejiang Province; Hangzhou, Zhejiang, China; 4 Division of Hepatobiliary and Pancreatic Surgery, First Affiliated Hospital, Zhejiang University School of Medicine, Hangzhou, Zhejiang, P.R. China

**Keywords:** portal vein tumor thrombus (PVTT), human umbilical vein endothelial cell (HUVEC), liver cancer, integrins, tumor microenvironment (TME), epithelial-mesenchymal transition (EMT)

## Abstract

**Background**: Liver cancer with portal vein tumor thrombus (PVTT) indicates a serious prognosis. The molecular mechanism of PVTT formation is not totally clarified, the invasion of blood vessels by liver cancer cells is the key step and portal vein endothelial cells plays critical role.

**Methods**: Conditioned medium (CM) of human umbilical vein endothelial cells (HUVEC) were used to culture liver cancer cells and prostate cancer cells for cell motility and viability analysis for the purpose of simulating the role of macrovascular endothelial cells in the development of liver cancer.

**Results**: HUVEC-CM caused long spindle-shaped changes in liver cancer cells; the invasion and migration ability of Bel-7402 and MHCC-LM3 (cultured in HUVEC-CM) increased significantly. Integrins/FAK (focal adhesion kinase) signaling pathway was activated and MMP-3 was up-regulated. However, classical epithelial-mesenchymal transition (EMT) did not involve. HUVEC-CM caused a decrease of cell population in G1- and S-phase of Bel-7402, it also caused an accumulation of cell population in G1 phase and a decrease of cell population in S-phase of MHCC-LM3, MHCC-97L and DU-145. HUVEC-CM promotes apoptosis of Bel-7402 and MHCC-97L and the nude mouse tumorigenic experiment did not find that the HUVEC-CM increase the tumorigenic ability of liver cancer cells.

**Conclusion**: HUVEC may provide an easy-to-adhere roadbed for liver cancer cells invasion of blood vessels by altering extracellular matrix (ECM), activating integrins/FAK pathway and inducing non-classical EMT. The effect of HUVEC-CM on cell viability was cancer cell type dependent. It is a meaningful glance at the mechsanism of PVTT.

## Introduction

Liver cancer is the third leading cause of cancer-related death worldwide 1, causing for over 600,000 deaths annually 2. Hepatocellular carcinoma (HCC) is the dominant form of primary liver cancer, accounting for more than 90% of all liver cancer cases 3. The early screening of cancer in China can improve the diagnosis and prognosis, but many patients were already in advanced stages on diagnosis because they have PVTT (the incidence is approximately 10-40%) 4. The prognoses of patients with liver cancer in the presence of PVTT is much poorer due to the comprehensive factors, such as impaired liver function, larger tumor load, reduced intolerance to anti-neoplastic therapy and complications related to portal hypertension, compared to patients without PVTT. It is reported that the overall survival rates have ranged from only 2 to 4 months after palliative care 5. Therefore, a thorough understanding of the mechanism of PVTT formation in liver cancer is helpful to improve the survival.

In recent years, tumor microenvironment (TME) has gained enormous attention. The TME is composed of cancer cells, combined with stromal cells and bioactive molecules they secrete. The hepatic stromal cells include vascular endothelial cells, sinusoidal endothelial cells, stellate cells, adipose cells, liver progenitor cells, immune inflammatory cells and cancer-associated fibroblasts. The crosstalk between liver cancer cells and immune inflammatory cells as well as cancer-associated fibroblasts significantly influences tumor growth and metastasis 6-9. It has also been documented that higher levels of activated hepatic stellate cells are associated to the increased portal vein invasion by up-regulation of integrins/FAK signaling pathway and MMP levels 10. Cancer-associated fibroblasts, tumor associated macrophages and endothelial cells are the main drivers of the ECM remodeling by secretion of a variety of enzymes. Besides, vascular endothelial cells are involved in the induction of EMT in various tumor types 11. During the process of the classical EMT, the epithelial markers including E-cadherin are decreased, but that of mesenchymal markers including N-cadherin are increased. EMT plays a critical role in the tumor progression via enhancing the motile and invasive activities and is significantly concerned with the generation of cancer stem-like cell population. Thus, we speculate that liver cancer cells enter into blood vascular systems following PVTT formation which is closely related to portal vein endothelial cells. However, the exact mechanism is elusive.

We used conditioned medium (CM) of HUVEC to culture liver cancer cells and prostate cancer cells in an attempt to disclose the role and mechanism of tumor-associated vascular endothelial cells in the PVTT formation in liver cancer.

## Materials and Methods

### Cell culture

The human prostate cancer cell DU-145 was a generous gift from Dr. Hong, Chen (Zhejiang University, China). HUVEC cell lines and Human hepatocellular carcinoma cell lines MHCC-LM3, MHCC-97L, Bel-7402 were purchased from Shanghai Cell Bank, Chinese Academy of Sciences. HUVEC cells were maintained in RPMI-1640 (Gibco) containing 10% FBS (fetal bovine serum) (Sigma-Aldrich) and incubated at 37°C in a 5% CO_2_ water-saturated environment. Conditioned medium of HUVEC cells (HUVEC-CM) was collected as previously described 12. RPMI-1640 medium supplemented with 10% FBS severed as the control medium. MHCC-LM3, MHCC-97L, Bel-7402 and DU-145 cells were respectively incubated in the HUVEC-CM for 21 days (n=3). MHCC-LM3 and MHCC-97L were subcultured once a week at a ratio of 1:2. Bel-7402, DU-145 and HUVEC were subcultured once a week at a ratio of 1:3 or 1:5.

### Transwell migration and invasion experiment

After 21 days MHCC-LM3, MHCC-97L, Bel-7402 and DU-145 were cultured in HUVEC-CM, the migration and invasion assays were performed using Transwell chambers with 8µm pore filters (Millipore, Billerica, MA, USA) as we previously described 12.

### Western blot analysis

Experimental cancer cells (after culturing in HUVEC-CM for 21 days) and respective control cells were lysed on ice in a lysis buffer (RIPA, Beyotime, Shanghai, China) with a protease inhibitor mixture cocktail (Roche, Switzerland). Western-blot analysis was performed by established protocols 12. Anti-α-catenin, anti-E-cadherin, anti-ZEB-1, ZEB-2, Snail, Slug, anti-ZO-1 and anti-Laminin A1 primary antibodies were purchased from (Abcam); anti-MMP-1, -2, -3, -11, -12, -13, -17 and -21, anti- ITGA6, B1, B3, B4, B7, anti-FAK, P-FAK-Y397, anti-Src, P-Src-Y418, P-Src-Y529, anti-fibronectin, anti-Laminin B3, anti-Wnt-2, anti-Wnt-5B, anti-Wnt-16, anti-TGF-β, anti-β-catenin and anti-N-cadherin primary antibodies were purchased from (Epitomics); anti-Cdc-2, P-Cdc-2-Tyr15, CDK4, CDK6, Cyclin A, Cyclin D1, Cyclin D3, Cyclin E2, P15, P16, P21, P27, P53, Rb, P-Rb-S811, P-Rb-S780; anti-Bcl-xl, Bad, P-Bad-Ser112, Bak, Mcl-1, Puma and anti-β-actin were from (Cell Signaling Technology).

### Confocal immunofluorescent analysis and cytoskeletal staining

Confocal immunofluorescent analysis and cytoskeletal staining were performed and analyzed as previous report 12, 13. Anti-vimentin and anti-γ-catenin primary antibodies were purchased from (Abcam); anti-Ezrin, anti-paxillin, anti-vitronectin and anti-P120 catenin primary antibodies were purchased from (Epitomics). Filamentous actin was stained with rhodamine-conjugated phalloidin (Sigma-Aldrich) in 1% bovine serum albumin in PBS for 30 minutes at 37°C.

### Cell-cycle analysis and cell apoptosis analysis

Cell-cycle analysis and cell apoptosis were conducted as previously described 12.

### Si-RNA based ITGB4 and ITGB7 knockdown

Si-RNAs directed against human ITGB4 and ITGB7 were purchased from Shanghai GenePharma. The siRNA sequences are shown as follow, Si-NC: Sense 5'-UUCUCCGAACGUGUCACGUTT-3', Anti-sense 5'-ACGUGACACGUUCGGAGAATT-3'; Si-ITGB4-4204: Sense 5'-GGUCACCUCCAAGAUGUUCTT-3'; Anti-sense 3'-GAACAUCUUGGAGGUGACCTT-5'; Si-ITGB4-5690: Sense 5'-GGACUGGGUCCUUUCACAUTT-3'; Anti-sense 3'-AUGUGAAAGGACCCAGUCCTT-5'; Si-ITGB7-5402: Sense 5'-GCUGAGUAAACUGAUUCCUAATT-3'; Anti-sense 3'-UUAGGAAUCAGUUUACUCAGCTT-5'; Si-ITGB7-5404: Sense 5'-ACCACCAUCAAUCCUCGCUUUTT-3'; Anti-sense 3'-AAAGCGAGGAUUGAUGGUGGUTT-5'. Transfection of the siRNA in Bel-7402 cells was conducted with Lipofectamine 2000 (Invitrogen) following the manufacturer's instructions.

### CO-IP experiment

Cells (MHCC-LM3 and Bel-7402) were lysed on ice in a lysis buffer (NP-40, Beyotime, Shanghai, China) with a protease inhibitor PMSF (Beyotime, Shanghai, China). After cell full lysis, 14 000 g centrifugation for 5 minutes. Take 300 ml protein sample and add 40 microliters Protein A+G Agarose. Slowly shake for 2 hours at 4°C. 1000g centrifugation for 5 minutes, extraction of supernatant for subsequent immunoprecipitation. Addition of 2 micrograms of the primary antibody for immunoprecipitation. Slowly shake overnight at 4°C. Then, add 40 microliters Protein A+G Agarose. Slowly shake for 3 hours at 4°C. 1000g centrifugation for 5 minutes. Carefully removed the supernatant. Then washed five times using PBS. Subsequent Western-blot analysis was then performed.

### Animal studies

Animal studies were conducted according to the Guide for the Care and Use of Laboratory Animals and with the approval of Institutional Animal Care and Use Committee. Tumor xenografts were generated by injection of 2 × 10^6^ experimental liver cancer cells (Bel-7402 and MHCC-97L cultured in HUVEC-CM cells for 21 days) and 2 × 10^6^ respective control cells into the subcutaneous tissue of the axillary region of nude mices, and mices were dissected 4 wk later (n = 5 mice/group). Tumor volume was calculated with the following formula: volume = π/6 × length × width2.

### Statistical analysis

Independent Student *t* test was used to analyze the differences between 2 groups. Statistical significance was accepted if *P* < 0.05. Statistical analysis was conducted using SPSS 16.0 software (SPSS).

## Results

### Cell morphology and capability of migration and invasion

**Figure 1:**
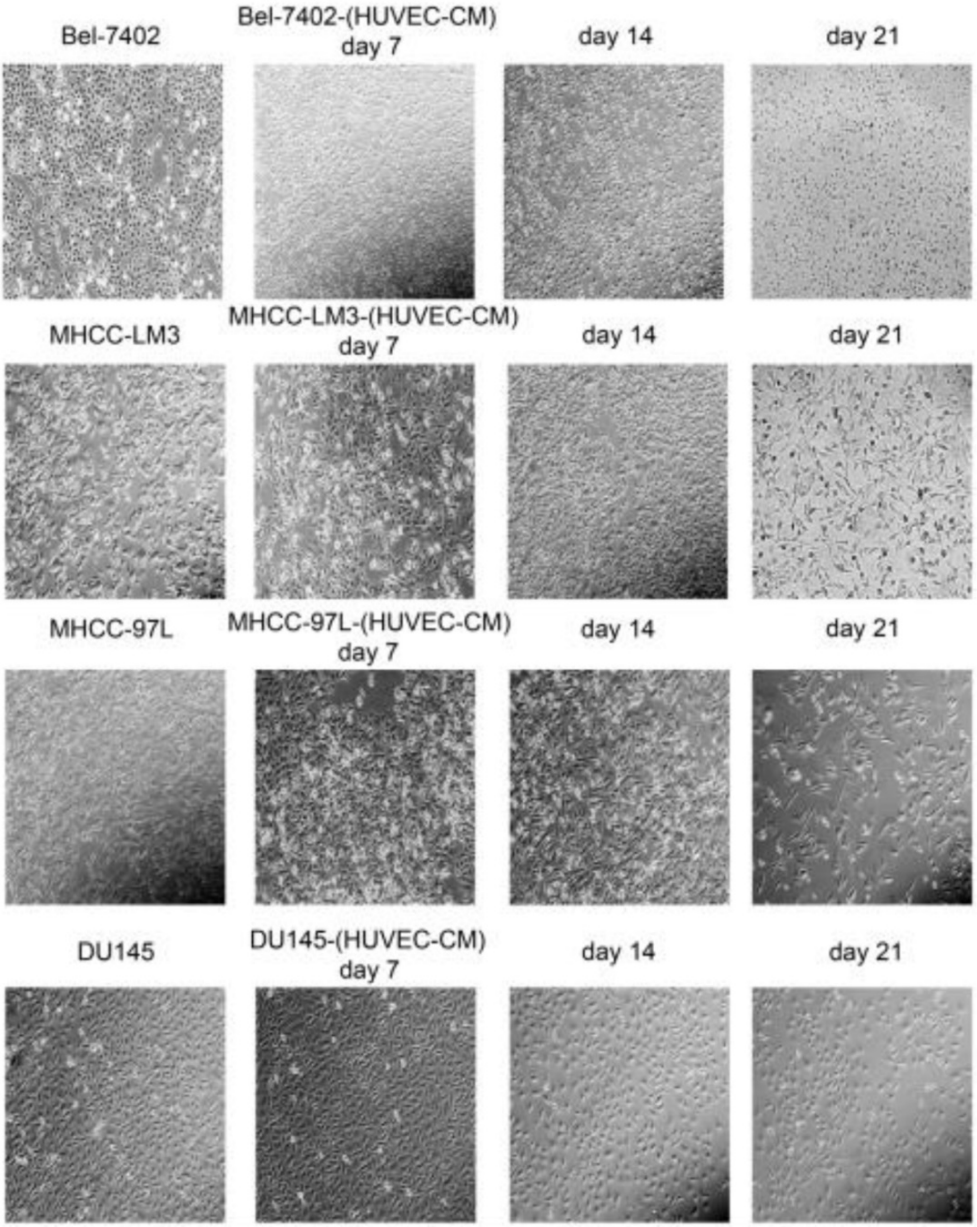
Morphological changes in prostate cancer cells and liver cancer cells after culture in HUVEC-CM for 21 days.

**Figure 2:**
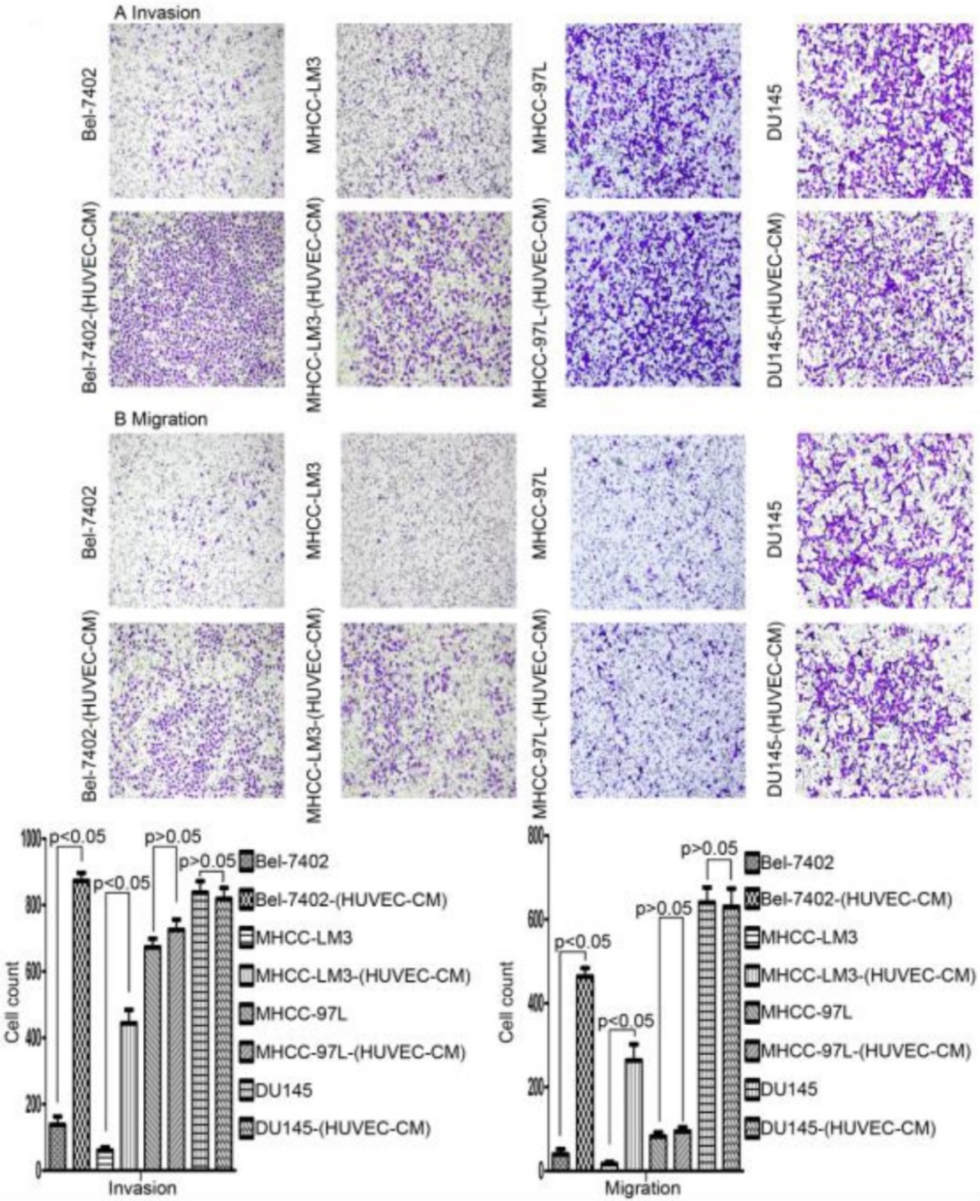
Alteration in cell motility. The invasion and migration ability of Bel-7402 and MHCC-LM3 cells cultured in HUVEC-CM for 21 days was enhanced relative to the control (*P*<0.05). However, the cell motility of MHCC-97L and DU-145 was not increased (*P*>0.05).

After culturing in HUVEC-CM for 21 days, liver cancer cells became obviously elongated. But, there was no significant change in cell morphology of prostate cancer cell DU-145 (Figure 1). The cell motility and invasiveness potentials of MHCC-LM3-(HUVEC-CM) and Bel-7402-(HUVEC-CM) were significantly augmented compared with control (*P*<0.05, Figure 2). However, the cell motility of MHCC-97L-(HUVEC-CM) and DU-145-(HUVEC-CM) were not enhanced (*P>*0.05, Figure 2).

### Expression of MMPs, EMT-related proteins, integrins/FAK/Src and laminins

**Figure 3:**
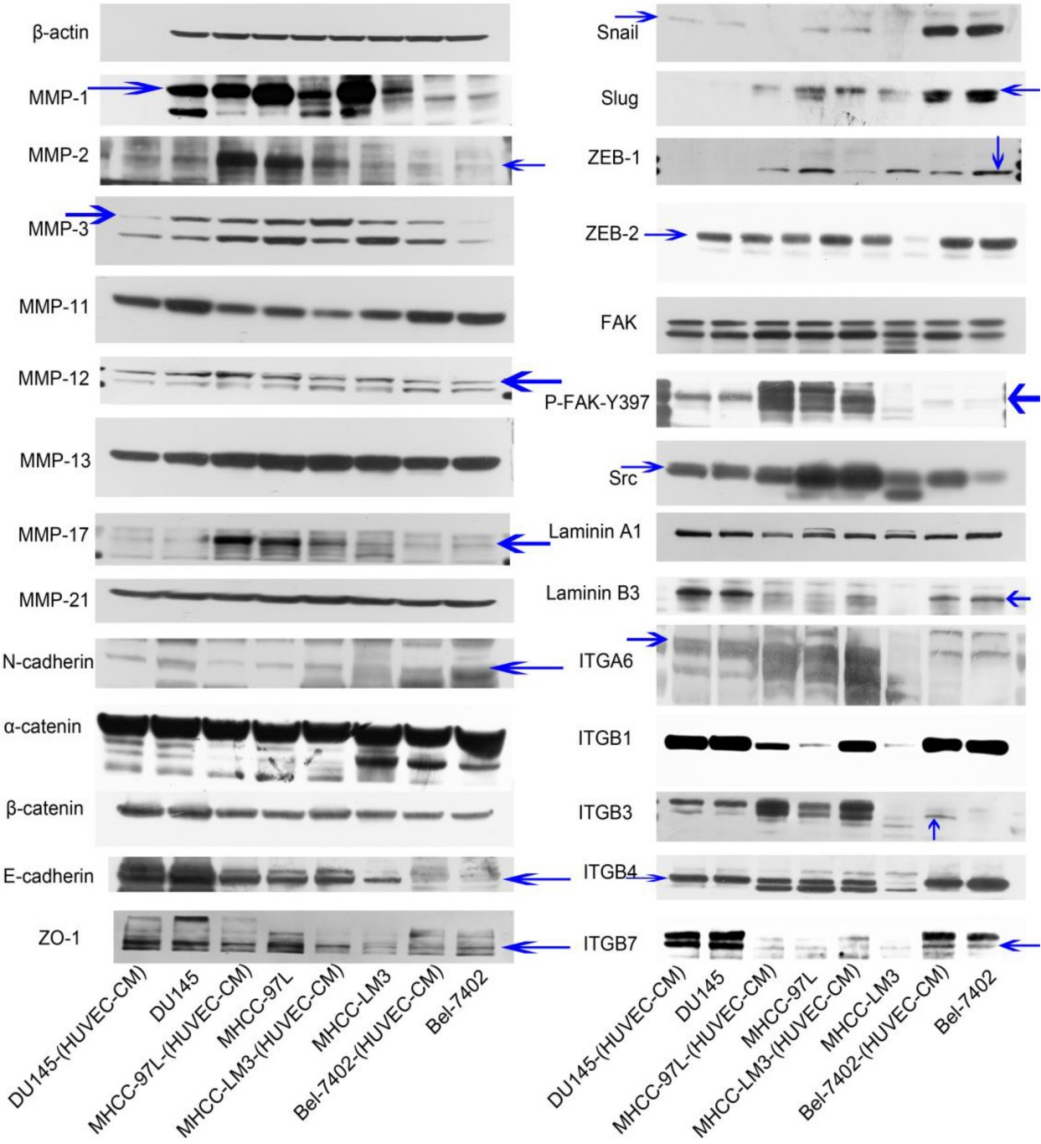
Alterations in expression profiles of epithelial markers, mesenchymal markers, EMT-related transcription factors, MMPs, laminins and integrins/FAK/Src signaling pathway.

To find out the mechanism of enhanced invasion and migration of MHCC-LM3-(HUVEC-CM) and Bel-7402-(HUVEC-CM), the expression profiles of epithelial markers E-cadherin, α-catenin and ZO-1; mesenchymal markers N-cadherin and β-catenin; EMT-related transcription factors Snail, Slug, ZEB-1, and ZEB-2; MMP-1, -2, -3, -11, -12, -13, -17, -21; integrins (ITGA6, B1, B3, B4, B7), FAK, P-FAK-Y397, Src and Laminin A1 and B3 were evaluated by Western-blot analysis (Figure 3). MMP-3, ITGB3, ITGB7, FAK, P-FAK-Y397 and Src were increased obviously in Bel-7402-(HUVEC-CM) compared with the control (Bel-7402). MMP-1, -2, -11, -12, -13, -17 and -21, E-cadherin, N-cadherin, α-catenin, β-catenin, ZO-1, Snail, Slug, ZEB-2, Laminin A1 and B3, ITGA6, B1 and B4 remained unchanged in Bel-7402-(HUVEC-CM) compared with the control. Whereas, EMT-related transcription factor ZEB-1 was reduced. MMP-1, -2, -3, -17, E-cadherin, N-cadherin, Snail, Slug, ZEB-2, FAK, P-FAK-Y397, Src, Laminin B3, ITGA6, B1, B3 and B4 were increased obviously in MHCC-LM3-(HUVEC-CM) compared with the control (MHCC-LM3). ITGB7 was increased moderately. MMP-12, 13 and -21, α-catenin, β-catenin, ZO-1 and Laminin A1 remained unchanged in MHCC-LM3-(HUVEC-CM) compared with the control. Whereas, EMT-related transcription factor ZEB-1 and MMP-11 were reduced. MMP-1,-17, ITGB1, B3 and B7 were increased in MHCC-97L-(HUVEC-CM) compared with the control (MHCC-97L). MMP-2, -3, -11, -12,-13, -21, E-cadherin, ZO-1, N-cadherin, α-catenin, β-catenin, FAK, P-FAK-Y397, Laminin A1 and B3, ITGA6 and B4 remained unchanged in MHCC-97L-(HUVEC-CM) compared with the control. On the other hand, the expressions of Snail, Slug, ZEB-1, ZEB-2 and Src were reduced. The above mentioned proteins were unchanged in DU-145-(HUVEC-CM) compared to control, except with reduction of MMP-3 and MMP-11 obviously. Collectively, these data indicate that MHCC-LM3-(HUVEC-CM) and Bel-7402-(HUVEC-CM) increase in cell motility through elevated expression of MMPs (especially MMP-3), integrins/FAK signaling pathway (The ratio discrepancy was listed in Additional files 1, 2, 3: Figure S1-3).

### Immunofluorescence results of epithelial and mesenchymal markers, cell motility-associated adhesion molecules and F-actin

**Figure 4:**
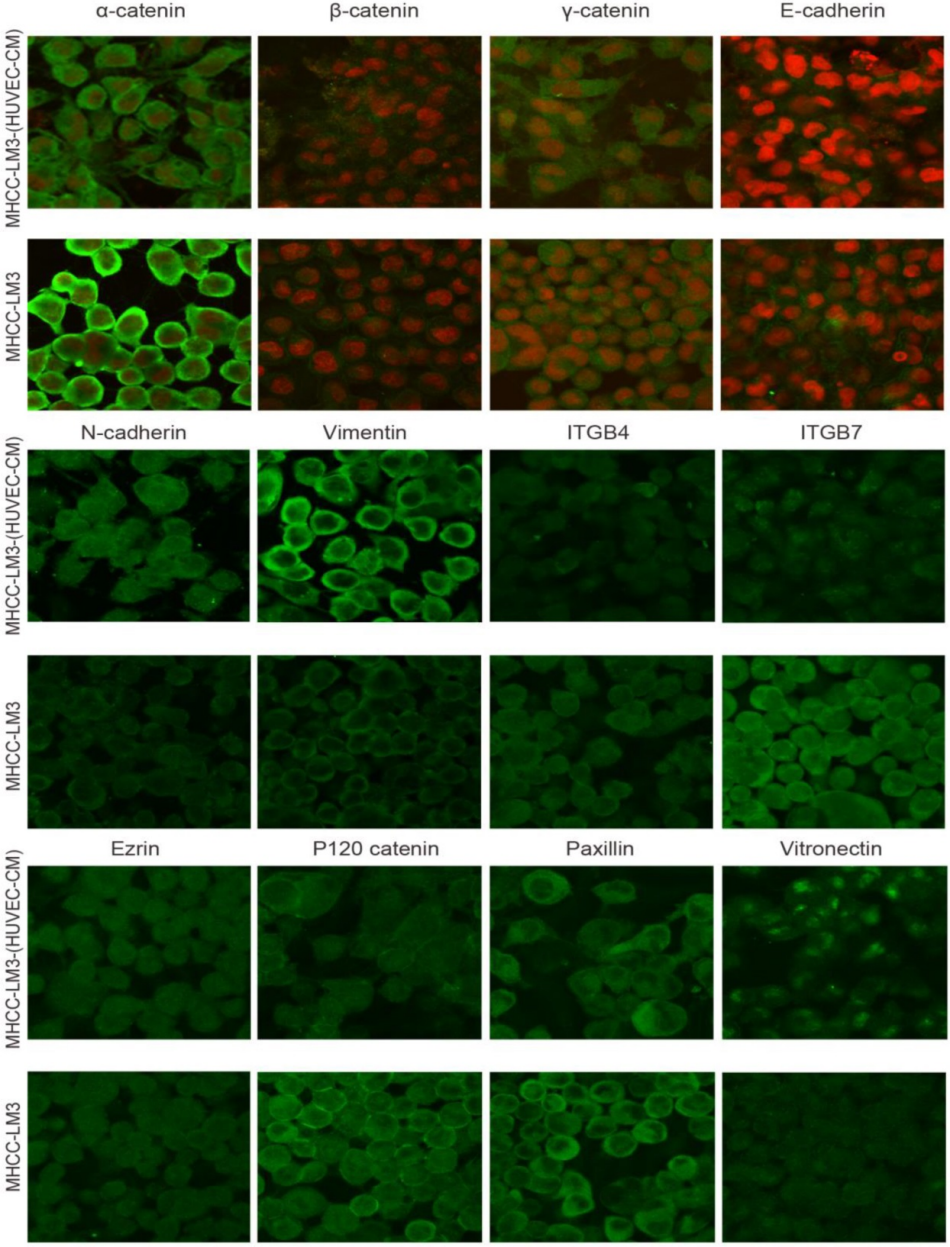
Immunofluorescence analysis of epithelial markers, mesenchymal markers, integrins and cell motility-associated adhesion molecules in MHCC-LM3 cells compared with MHCC-LM3-(HUVEC-CM) cultured in HUVEC-CM for 21 days.

**Figure 5:**
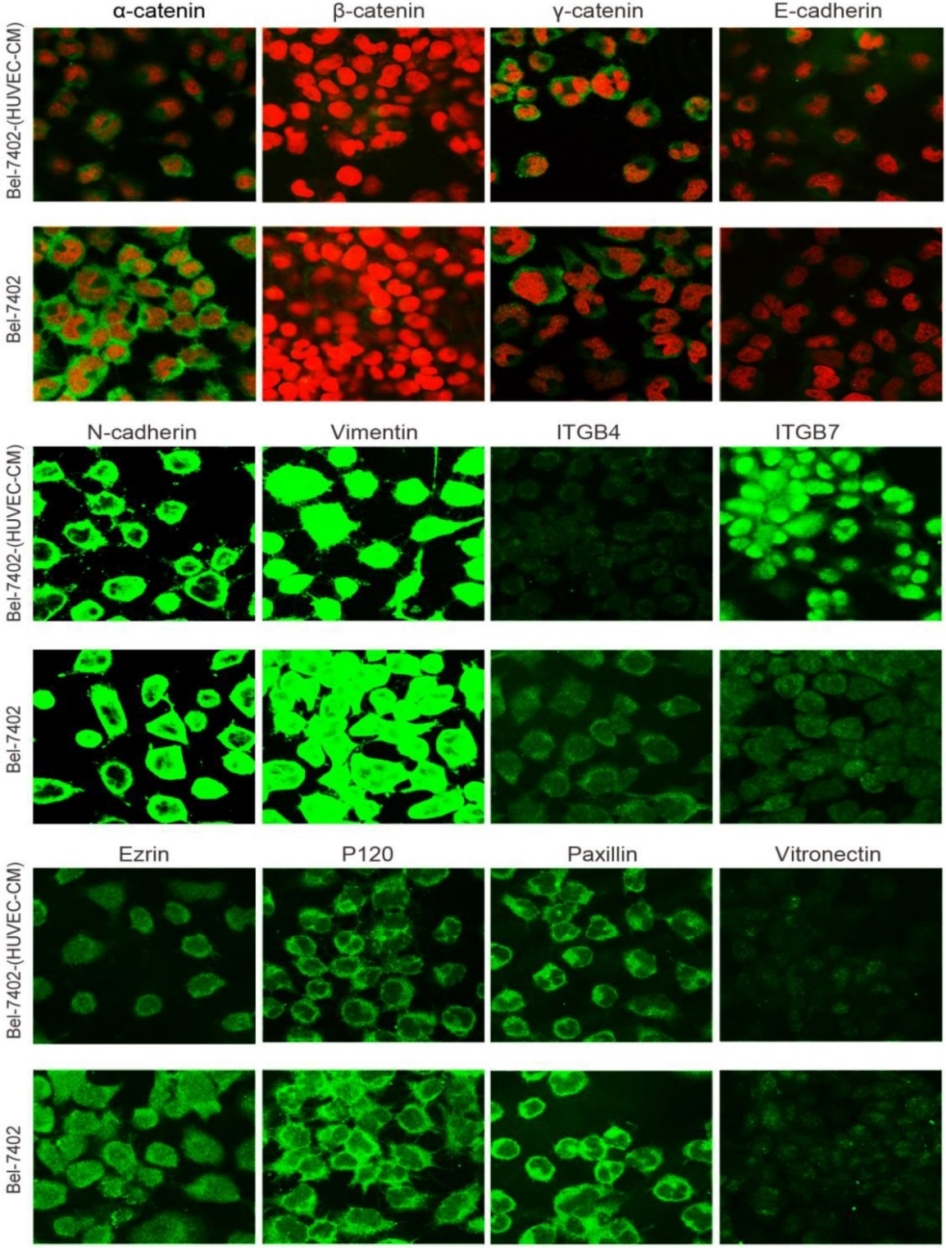
Immunofluorescence analysis of epithelial markers, mesenchymal markers, integrins and cell motility-associated adhesion molecules in Bel-7402 cells compared with Bel-7402-(HUVEC-CM) cultured in HUVEC-CM for 21 days.

**Figure 6:**
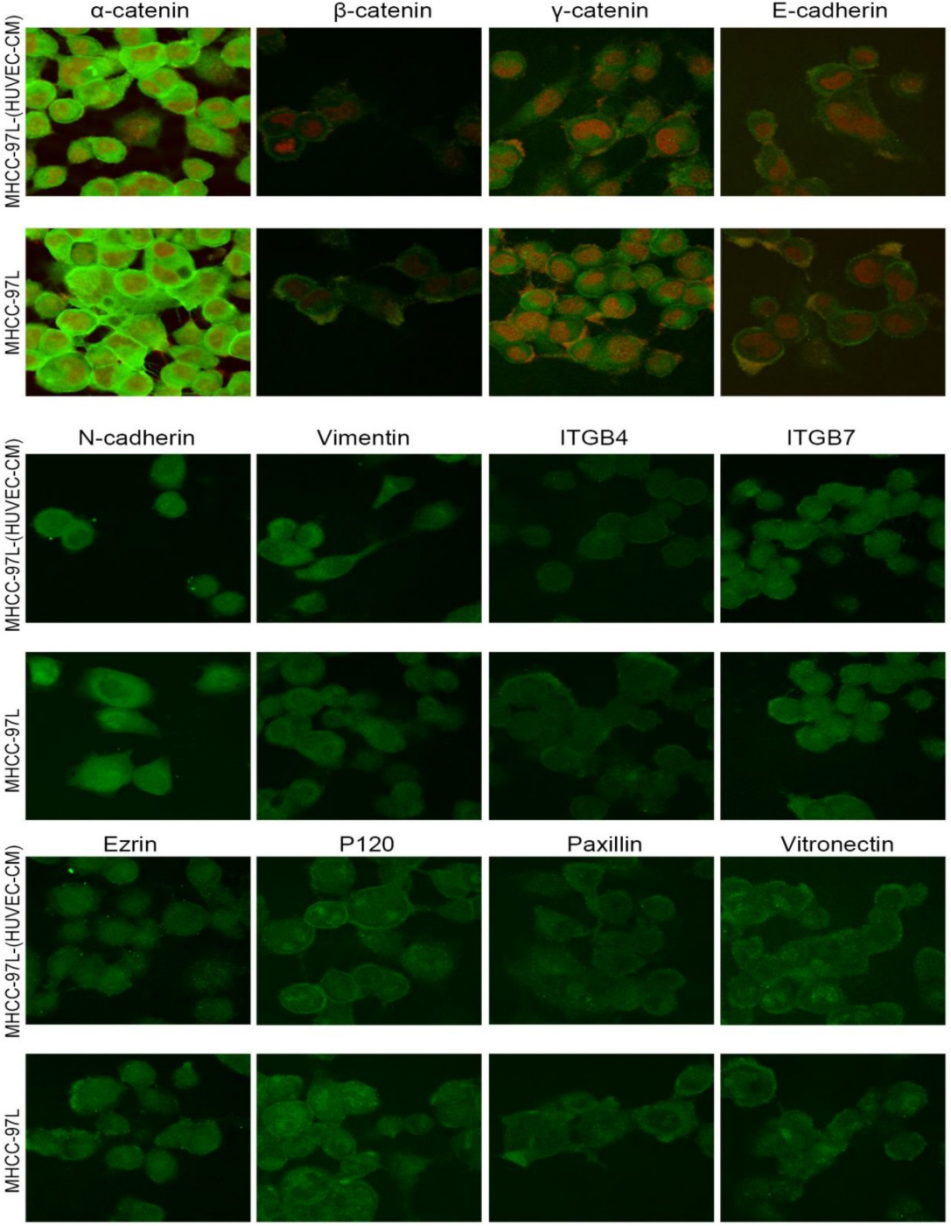
Immunofluorescence analysis of epithelial markers, mesenchymal markers, integrins and cell motility-associated adhesion molecules in MHCC-97L cells compared with MHCC-97L-(HUVEC-CM) cultured in HUVEC-CM for 21 days.

**Figure 7:**
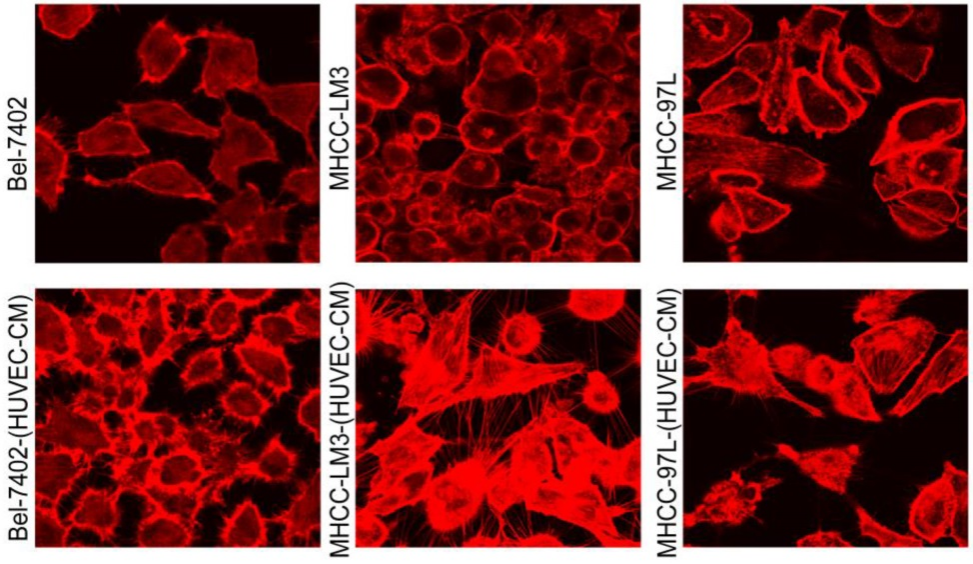
F-actin evaluation. Bel-7402 and MHCC-LM3 cells cultured in HUVEC-CM cells for 21 days had more actin-rich protrusions.

To further determine the mechanisms of enhanced cell invasion and migration in MHCC-LM3-(HUVEC-CM) and Bel-7402-(HUVEC-CM), immunofluorescence analysis was performed. The expressions of α-catenin and P120-catenin on cell membrane were significantly reduced in MHCC-LM3-(HUVEC-CM) relative to the control (Figure 4). The connection of β-catenin and E-cadherin on cell membrane tends to be unstable. Vimentin and N-cadherin were increased dramatically. ITGB7 and vitronectin tends to cluster inside of cells. However, γ-catenin, ITGB4, Ezrin and paxillin were unchanged. The expression of α-catenin on cell membrane were significantly reduced in Bel-7402-(HUVEC-CM) relative to the control (Figure 5). The connection of β-catenin on cell membrane tends to be unstable, too. Vimentin and ITGB7 were increased dramatically and ITGB7 tends to cluster inside of cells. However, γ-catenin, P120-catenin, E-cadherin, N-cadherin, ITGB4, Ezrin, paxillin and Vitronectin were unchanged. We did not find such significant changes, as mentioned above, in MHCC-97L-(HUVEC-CM) compared to controls (Figure 6). Moreover, filamenttous actin (F-actin), which is essential for cell motility, was increased in MHCC-LM3-(HUVEC-CM) and Bel-7402-(HUVEC-CM), while with no obvious change in MHCC-97L-(HUVEC-CM) compared to controls (Figure 7). The results suggest that Bel-7402-(HUVEC-CM) and MHCC-LM3-(HUVEC-CM) were induced to undergo non-classical EMT via redistribution of E-cadherin/catenin complex and up-regulation of vimentin and N-cadherin. The results also verified that integrins may play an indispensable role in the enhanced motility of liver cancer cells (The fluorescence intensity comparison was showed in Additional files 4, 5: Fig. S4, 5).

### Effect of HUVEC-CM on cell viability

**Figure 8:**
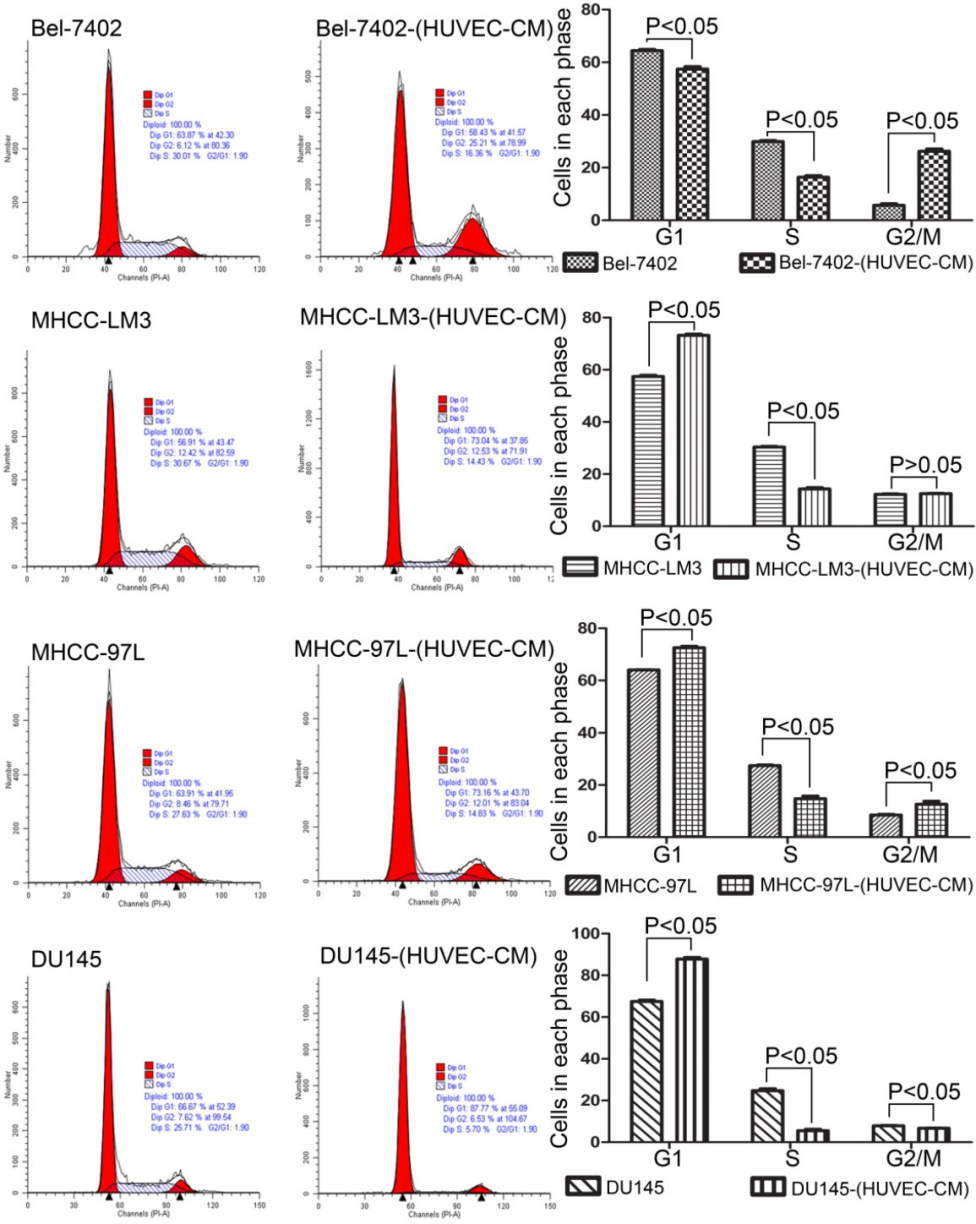
Cell cycle profiles of prostate cancer cells and liver cancer cells after culture in HUVEC-CM for 21 days were evaluated by flow cytometry.

**Figure 9:**
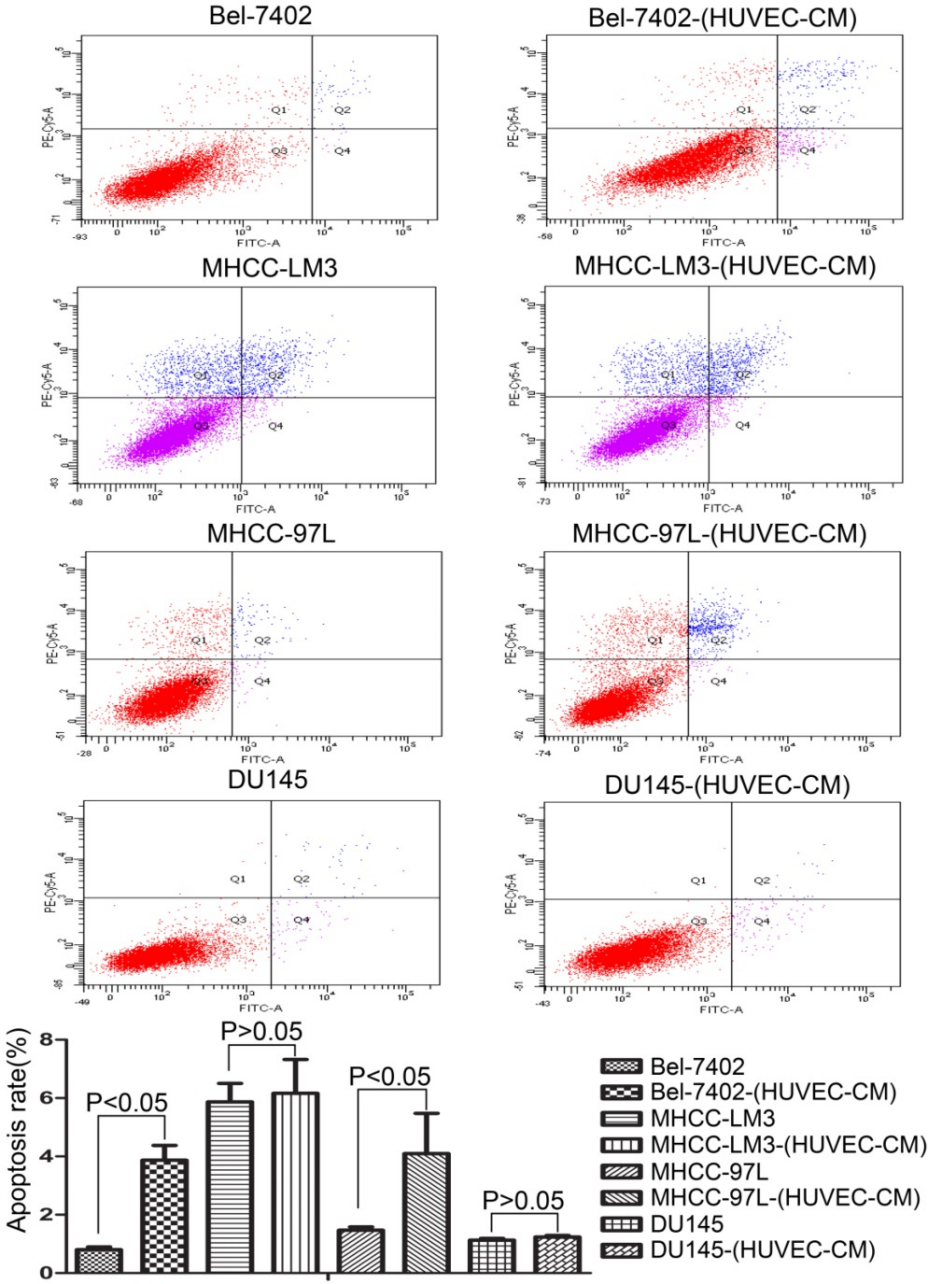
Apoptotic rate of prostate cancer cells and liver cancer cells after culture in MRC-5-CM for 21 days were evaluated by flow cytometry.

To explore the effect HUVEC-CM on cancer cell growth, we used flow cytometry to investigate the change of cell cycle progression and apoptosis. We found that HUVEC-CM caused a decrease of cell population in G1- and S-phase, along with an increase of G2/M cells. HUVEC-CM caused an accumulation of cell population in G1 phase of MHCC-LM3, MHCC 97L and DU145, along with a concomitant decrease of cell population in S-phase (Figure 8). HUVEC-CM promotes apoptosis of Bel-7402 and MHCC97L (Figure 9).

**Figure 10:**
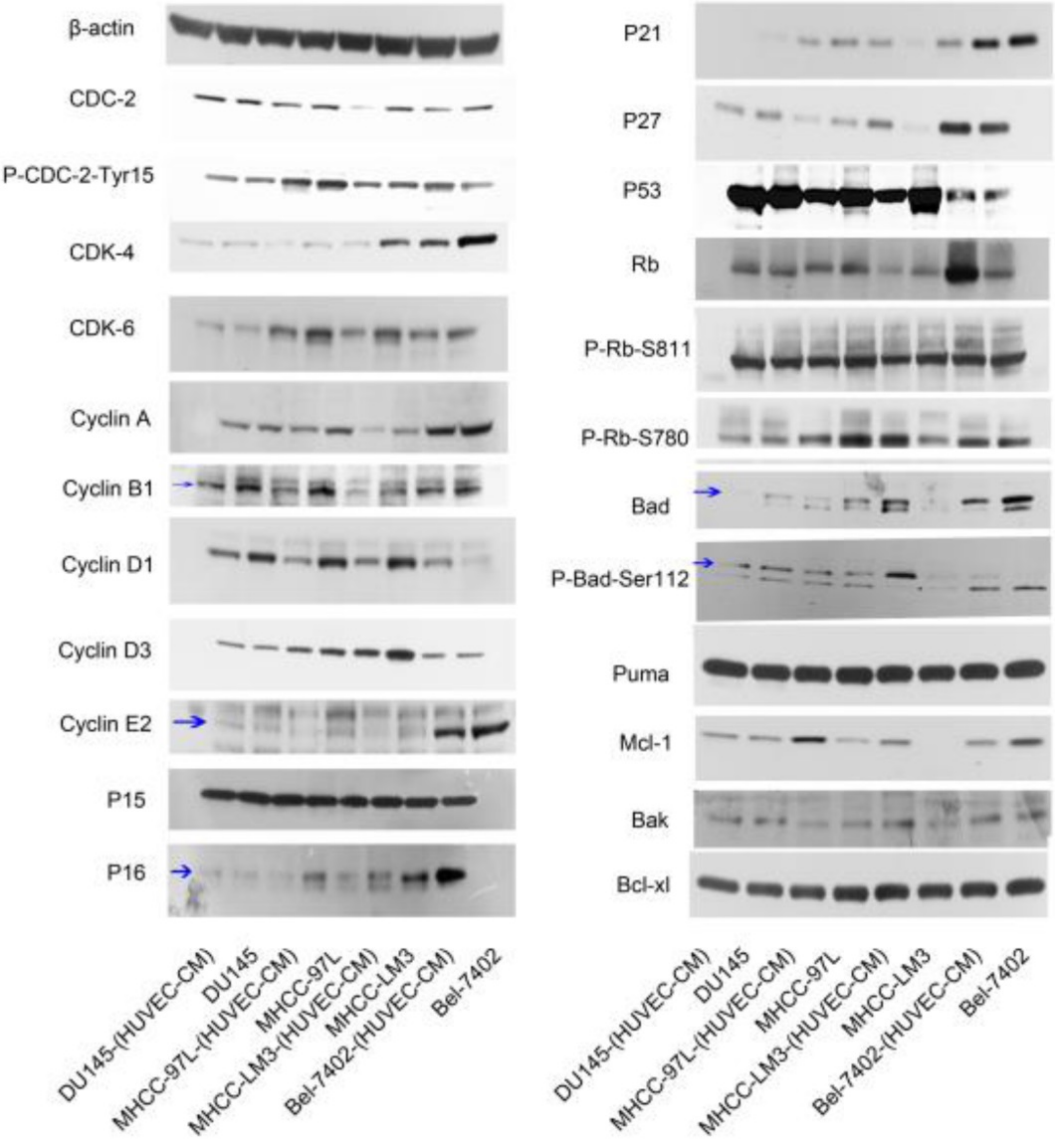
Alterations in expression profiles of cell cycle and cell apoptosis-related proteins.

To have a better understanding of the effect of HUVEC-CM on cell cycle process, we detected the expression of cell cycle-related proteins (Figure 10). Cdc-2, which permits the entry of cells from G2 phase into M phase, was decreased in Bel-7402-(HUVEC-CM), but Tyr15-phosphorylated Cdc-2 (results in Cdc-2 inhibition), was increased. CDK-4 which are essential for the transition from G1 to S phase, were reduced in Bel-7402-(HUVEC-CM), compared to control. CDK-6 was unchanged. Cyclin A (S phase protein), B1 (G2/M phase protein), D3 and E2 (G1 phase protein) was unchanged. Cyclin D1 (G1 phase protein) was increased. P15, P21, P27 and P53, which inhibit cells in G1 phase from entering S phase, remained unchanged in Bel-7402-(HUVEC-CM), compared to control. However, P16 was down-regulated. Rb which promotes G1-S phase progression, was increased. Phosphorylated Rb (P-Rb-S811 and P-Rb-S780) was unchanged. Cdc-2 was decreased in MHCC-LM3-(HUVEC-CM), but Tyr15-phosphorylated Cdc-2, was unchanged, compared to control. CDK-4 and CDK-6 were reduced. Cyclin A, B1, D1, D3 and E2 were decreased. P15 and P53 remained unchanged. P16 and P21 were down-regulated. However, P27 was up-regulated. Rb and P-Rb-S811 were unchanged. P-Rb-S780 was increased. Cdc-2 was decreased in MHCC-97L-(HUVEC-CM), but Tyr15-phosphorylated Cdc-2 was unchanged compared to the control. CDK-4 was decreased. CDK-6 remained unchanged. Cyclin A, B1, D1 and E2 were decreased in varying content. Cyclin D3 was unchanged. P15 and P53 remained unchanged. P16, P21 and P27 were down-regulated. Rb and P-Rb-S811 were unchanged. P-Rb-S780 was decreased. Interestingly, we only found Cyclin D1 and p21 were decreased in DU-145-(HUVEC-CM), compared to control. We need to further explore the mechanism of HUVEC affecting the cell cycle transition of cancer cells.

The expression profiles of apoptosis-associated proteins were also evaluated (Figure 10). The pro-apoptotic Bad gene was decreased in Bel-7402-(HUVEC-CM), compared to control, but phosphorylated Bad (P-Bad-Ser112), which inhibited the apoptotic activity of Bad, was increased. Pro-apoptotic gene Puma was unchanged. Anti-apoptotic Mcl-1 gene was reduced. Anti-apoptotic genes Bak and Bcl-xl were unchanged. Bad, P-Bad-Ser112 and Mcl-1 were increased in MHCC-LM3-(HUVEC-CM), compared to control. Puma, Bak and Bcl-xl were unchanged. Bad was decreased in MHCC-97L-(HUVEC-CM), compared to control. However, Mcl-1 was increased. P-Bad-Ser112, Puma and Bcl-xl were unchanged. Bak was increased. Bad and P-Bad-Ser112 was down-regulated in DU-145-(HUVEC-CM), compared to control. Puma, Mcl-1, Bak and Bcl-xl were unchanged. We need to further explore the specific mechanism of apoptosis of tumor cells induced by HUVEC-CM. (The ratio discrepancy was listed in Additional files 6, 7: Figure S6, 7).

### Effect of HUVEC-CM on tumorigenicity

**Figure 11:**
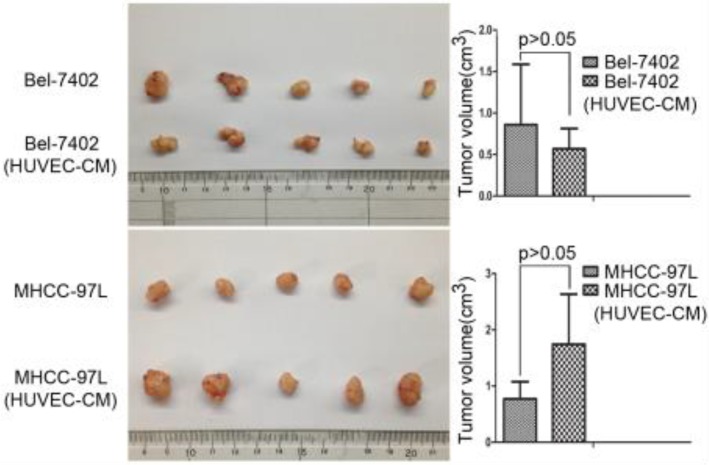
Tumor forming ability of Bel-7402 cells and MHCC-97L cultured in CM of HEVEC cells for 21 days were assessed in nude mice by axillary subcutaneous implant (n = 5 per grou**p**).

To determine the role of HUVEC in tumor development, we conducted nude mouse tumorigenicity experiments (Figure 11). Tumor xenograft studies revealed that HUVEC had no obvious effect on the tumorigenic ability of Bel-7402 and MHCC-97L.

### The role of ITGB4 and ITGB7 in liver cancer cells

**Figure 12:**
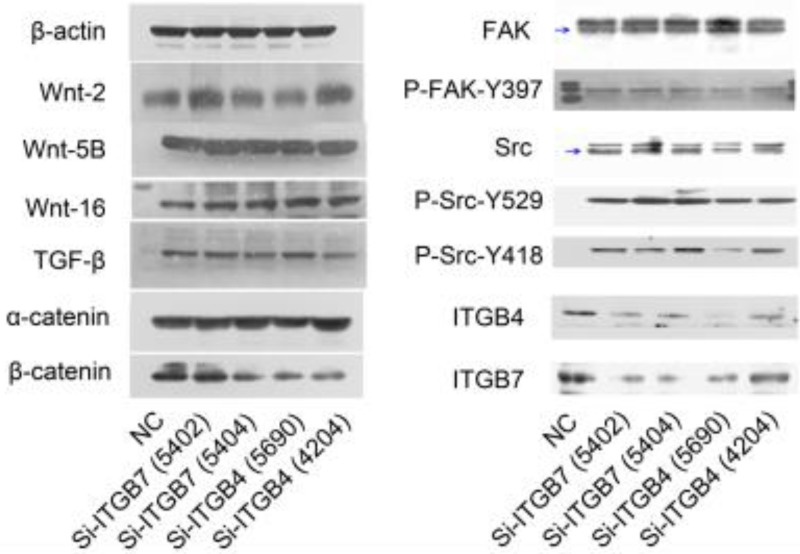
Alterations in the expression profiles of WNTs, TGF-β and FAK/Src signaling pathway in Bel-7402 cells after knockdown of ITGB4 and ITGB7.

**Figure 13:**
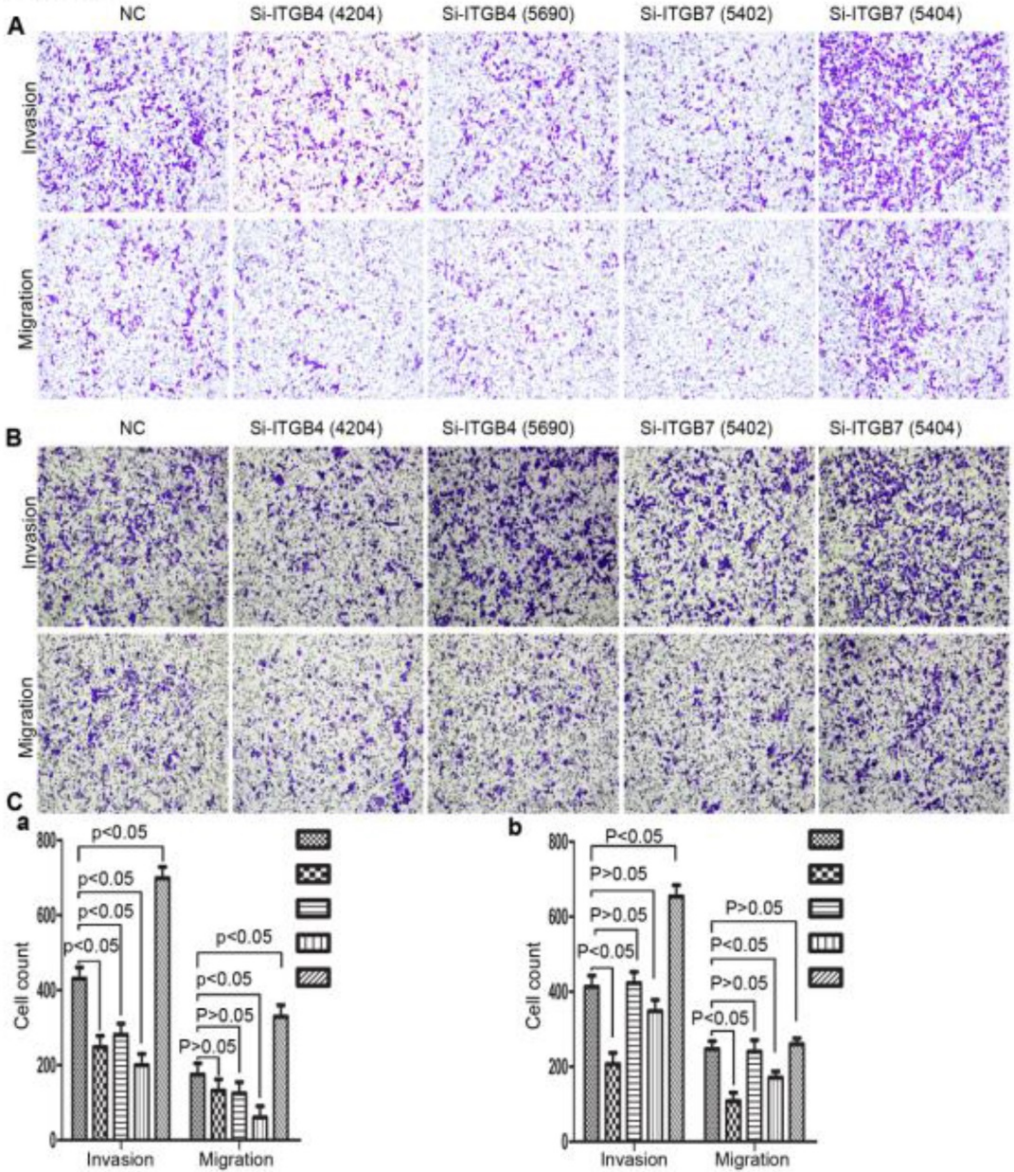
Alterations in cell motility of MHCC-97L (A) and MHCC-LM3 (B) after knockdown of ITGB4 and ITGB7.

**Figure 14:**
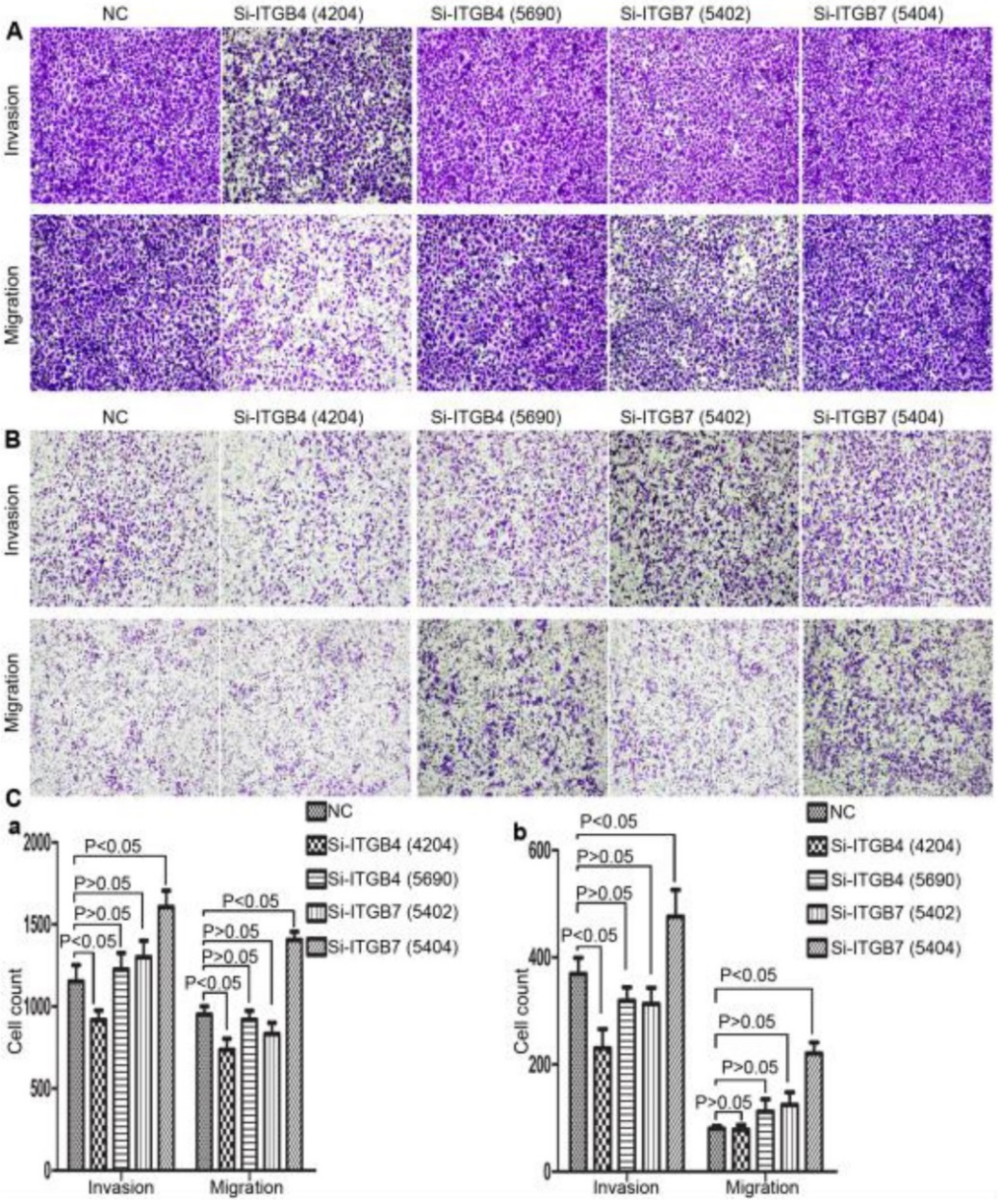
Alterations in cell motility of Bel-702 (A) and DU-145 (B) after knockdown of ITGB4 and ITGB7.

**Figure 15:**
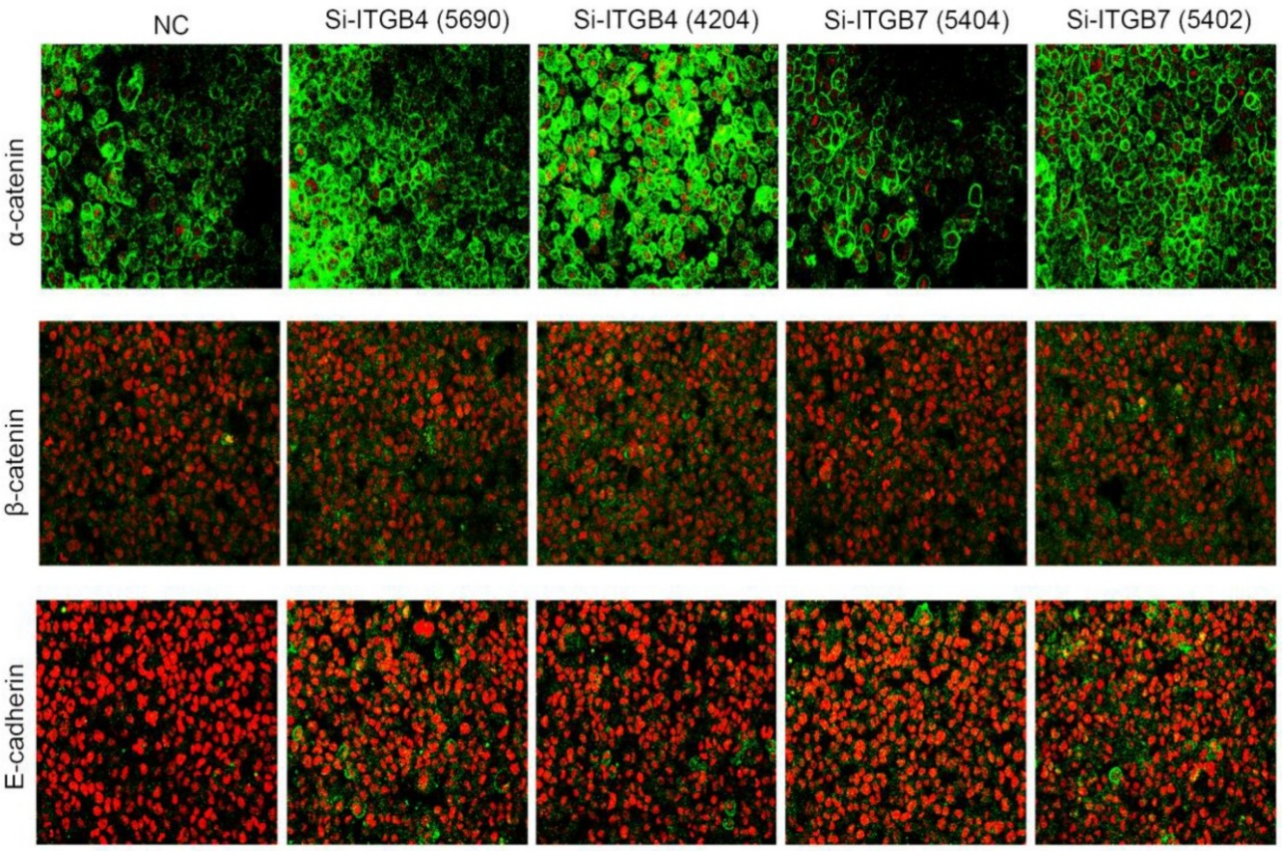
Immunofluorescence analysis of cell membrane expression of E-caderin, α-catenin and β-cadherin after knockdown of ITGB4 and ITGB7 in Bel-702.

**Figure 16:**
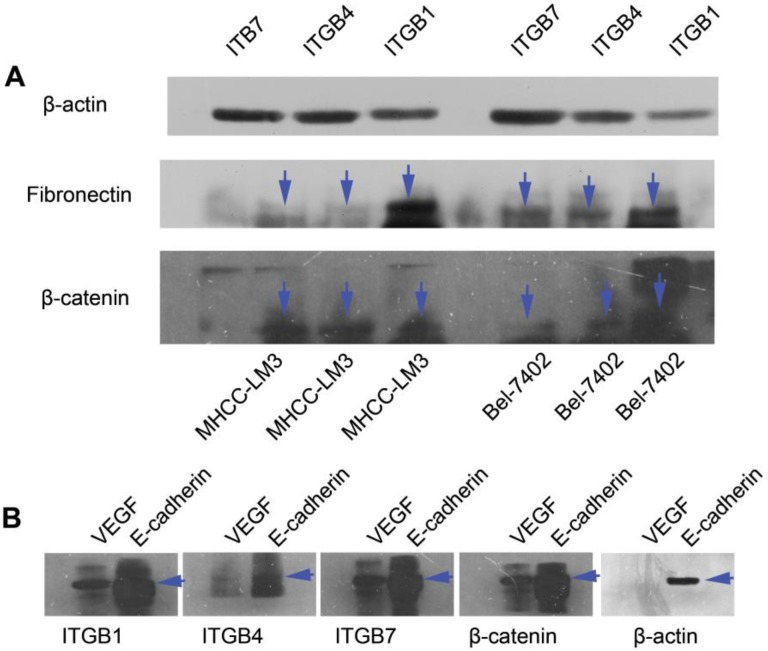
CO-IP analysis. (A) Primary antibodies for immunoprecipitation were ITGB1, ITGB4 and ITGB7. CO-IP assays show that ITGB1, ITGB4 and ITGB7 can bind with β-actin, β-catenin and fibronectin in MHCC-LM3 and Bel-7402 cells. (B) Primary antibodies for immunoprecipitation were ITGB1, ITGB4, ITGB7, β-actin and β-catenin. CO-IP assays show that β-catenin, ITGB1, ITGB4 and ITGB7 can bind with E-cadherin and VEGF. β-actin can bind with E-cadherin in MHCC-LM3 cells.

To determine the exact role of ITGB4 and ITGB7 in development of liver cancer, we used Si-ITGB4-4204 and Si-ITGB4-5690 to knockdown ITGB4, and Si-ITGB7-5402 and Si-ITGB7-5404 to knockdown ITGB7 gene in Bel-7402. The expression of Wnt-2, Wnt-5B, Wnt-16, α-catenin, β-catenin and P-Src-Y529 (inactivate Src) were unchanged in Si-ITGB4-4204, Si-ITGB4-5690, Si-ITGB7-5402 and Si-ITGB7-5404, compared to Si-NC. TGF-β was decreased slightly in Si-ITGB4-4204. The expression of FAK and P-FAK-Y397 (which is correlated to the activation of FAK), were increased slightly in Si-ITGB4-5690, Si-ITGB7-5402 and Si-ITGB7-5404, compared to Si-NC. Src and P-Src-Y418 (activate Src) was down-regulated in Si-ITGB4-5690, compared to control. Interestingly, we found that knockdown of ITGB7 also reduces the expression of ITGB4 (Figure 12). The ratio discrepancy was listed in additional file 8: Fig S8. Transwell assay results showed that Si-ITGB4-4204 weakened liver cancer cells motility significantly, but Si-ITGB7-5404 enhanced liver cancer cells motility evidently (Figure 13, 14). Immunofluorescence results showed that knockdown of ITGB4 and ITGB7 can up-regulated the expression of E-cadherin/catenin complex on membrane of liver cancer cells (Figure 15). The fluorescence intensity comparison was showed in additional file 9: Fig. S9. CO-IP results verified that ITGB1, B4 and B7 can bind with β-actin, β-catenin, VEGF, fibronectin and E-cadherin (Figure 16).

## Discussion

Liver cancer with PVTT is intractable and lethal. Although there are a variety of treatments, including molecular targeted therapy, liver resection, trans-arterial chemoembolization, radiotherapy, hepatic artery infusion chemotherapy or systemic chemotherapy, radiofrequency ablation and combination therapy, the overall survival rate remains unsatisfactory 14. Deeply understanding the biological behavior of liver cancer cells that are prone to invade blood vessels is the key to breakthrough in treatment.

The reciprocal crosstalk between “abnormal” cells and the surrounding microenvironment is a vicious circle leading to tumorigenesis and progress. Nevertheless, the TME is extremely complex and dynamically variable. The further development of tumors seems to be due to the dynamic variability of TME and it makes the treatment of cancer more complex and difficult. Tumor associated vascular endothelial cells is a main component of TME. Basically, proliferation of cancer cells requires blood vessels to provide oxygen and nutrition. Into the bargain, long-distance metastasis of tumors requires a passage like blood vessels and circulating tumor cells are the fundamental cause of cancer's unkilling and rebirth. In fact, it is evidenced that tumor-derived vascular endothelial cells are different from tumor associated vascular endothelial cells 15. But, apart from the difference between tumor-derived vascular endothelial cells and tumor associated vascular endothelial cells, we cannot ignore the role of tumor-associated vascular endothelial cells in tumors.

During our research, we found that HUVEC-CM caused long spindle-shaped changes in liver cancer cells obviously and enhanced the invasion and migration ability of Bel-7402 and MHCC-LM3. However, HUVEC-CM did not enhance the invasion and migration ability of MHCC-97L and DU-145. We thought it is due to up-regulation of MMP-3, by activation of integrins/FAK signaling pathway, and by inducing non-classical EMT. Western-blot results showed that MMP-1, -2, -3, -17 were up-regulated in MHCC-LM3-(HUVEC-CM), compared to control. MMP-3 was up-regulated in Bel-7402-HUVEC-CM, compared to control. It also showed that ITGA6, Β1, B3, B4 and B7 were increased in MHCC-LM3-(HUVEC-CM), compared to control. Western-blot results showed that ITGB3 and B7 were increased in Bel-7402-(HUVEC-CM), compared to control. Immunofluorescence test showed that ITGB7 was increased in Bel-7402-(HUVEC-CM), compared to control. It has been documented that activated integrin-FAK-Src functions to promote cell-cell and cell-matrix adhesion, cell migration and invasion 16. Western-blot results showed that FAK and Src were both increased in MHCC-LM3-(HUVEC-CM) and Bel-7402-(HUVEC-CM) compared to controls. This is an important result, because the expression of these two proteins has not increased in MHCC-97L-(HUVEC-CM) and DU-145-(HUVEC-CM), which can explain why the invasion and migration ability has not increased. Classical EMT decreases the expression of e-cadherin, which is the increase of N-cadherin and β-catenin expression. Western-blot and immunofluorescence test did not show that β-catenin was increased obviously in MHCC-LM3-(HUVEC-CM) and Bel-7402-(HUVEC-CM) compared to controls. Western-blot and immunofluorescence test showed that N-cadherin was up-regulated in MHCC-LM3-(HUVEC-CM), compared to control. However, western-blot results also showed E-cadherin was up-regulated in MHCC-LM3-(HUVEC-CM), compared to control. Western-blot and immunofluorescence test did not show that N-cadherin was up-regulated or E-cadherin was down-regulated in Bel-7402-(HUVEC-CM) compared to control. Nevertheless, we found that the membrane expression of α-catenin and P-120 catenin was down-regulated in MHCC-LM3-(HUVEC-CM), compared to control. The membrane expression of α-catenin in Bel-7402-HUVEC-CM was also down-regulated, compared to control. In contrast, vimentin was up-regulated obviously in MHCC-LM3-(HUVEC-CM) and Bel-7402-(HUVEC-CM), compared to controls. Immunofluorescence test did not find such results in MHCC-97L. Thus, we confirmed that HUVEC-CM induced Bel-7402 and MHCC-LM3 to undergo non-classical EMT. Of course, we need to further explore how the vascular endothelial cells promote the tumor cells to “roll” in a designated route.

Integrins are heterodimeric integral membrane glycoproteins composed of α- and β-subunits forming 24 heterodimers. Different integrins are involved in different cellular processes, such as cell attachment to ECM and cell motility 17, 18. In this paper, we used siRNA technology to knockdown ITGB4 and B7 to reveal their role in liver cancer. We found that ITGB4 and B7 affect the motility of liver cancer cells. Si-ITGB4-4204 weakened liver cancer cells motility significantly, but Si-ITGB7-5404 enhanced liver cancer cells motility evidently. Surprisingly, it seems like that the more obvious the silencing of ITGB4 is, the less the migration and motility of cancer cells will be impaired, and the more obvious the silencing of ITGB7 is, the stronger the migration and movement ability of cancer cells is. What's more, the expression of FAK-Src pathway was not be inhibited obviously. Interestingly, we found that silencing ITGB7 also reduces the expression of ITGB4. Immunofluorescence test showed that silencing ITGB4 and ITGB7 can increase the membrane expression of α-cateninin, β-catenin and E-cadherin in liver cancer cells to some extent. CO-IP results also showed that ITGB1, ITGB4 and ITGB7 can bind to β-actin, β-catenin, VEGF, fibronectin and E-cadherin. We need to further explore the role of integrins in the occurrence and development of liver cancer. This result also gives us an inspiration that the therapeutic effect of antineoplastic drugs seems to depend on a balanced state, like gene silencing, silence is part of it, but it shouldn't be completely silent.

Vascular endothelial cells affect the tumor-specific cell cycle and apoptotic process. However, the nude mouse tumorigenic experiment did not find that the HUVEC-CM enhanced the tumorigenic ability of liver cancer cells. Existing cell cycle-related proteins and the Bcl-2 gene family cannot explain our cell cycle and apoptosis experimental results very well. Specific molecular mechanisms need to be further explored.

To conclude, HUVEC influences multiple pathways regulating invasion, migration, proliferation, and apoptosis in liver cancer. Anti-tumor angiogenesis molecular targeted therapy may require additional targets for oncogenic integrins.

## Supplementary Material

Supplementary figures.
