# Supplementary material for: Macrovascular Endothelial Cells Enhance the Motility of Liver Cancer Cells by Up-regulation of MMP-3, Activation of Integrin/FAK Signaling Pathway and Induction of Non-classical Epithelial-mesenchymal Transition
Source: J Cancer. 2020 Feb 3;11(8):2044–59. doi: 10.7150/jca.38209 (PMC7052939; doi:10.7150/jca.38209)
Supplement: Supplementary file 1 — Supplementary figures. [file jcav11p2044s1.pdf]

Supporting legends

Fig. S1-3. Ratio discrepancy of the target proteins/β-actin between prostate cancer and liver cancer cells cultured in HUVEC-CM and control cells.

Fig. S4. Fluorescence intensity comparison of EMT-related proteins, ITGB4, ITGB7, Ezrin, Paxillin and Vitronectin between liver cancer cells cultured in HUVEC-CM and liver cancer cells.

Fig. S5. Fluorescence intensity comparison of F-actin between liver cancer cells cultured in HUVEC-CM and liver cancer cells.

Fig. S6-7. Ratio discrepancy of the cell cycle and cell apoptosis associated proteins/β-actin between prostate cancer and liver cancer cells cultured in HUVEC-CM and control cells.

Fig. S8. Ratio discrepancy of WNTs, α-catenin, β-cadherin, FAK/Src signaling pathway and TGF-β/β-actin between Bel-7402 after knockdown of ITGB4/ITGB7 and Bel-7402.

Fig. S9. Fluorescence intensity comparison of cell membrane expression of E-cadherin, α-catenin and β-catenin between Bel-7402 after knockdown of ITGB4/ITGB7 and Bel-7402.

Fig. S1

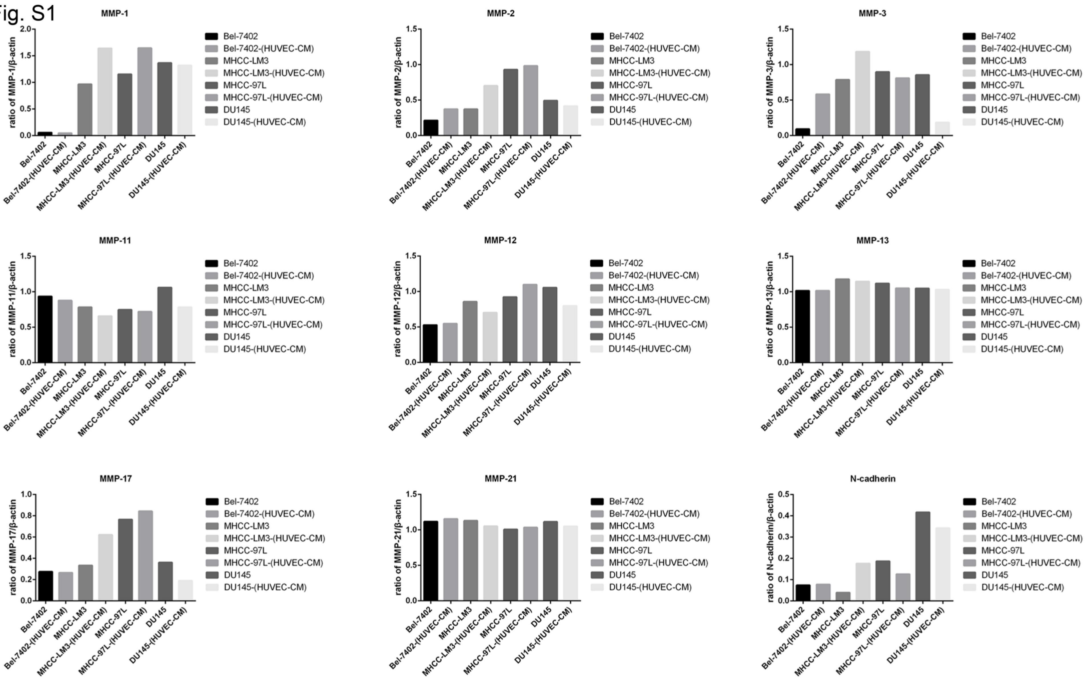
MMP-1
ratio of MMP-1/β-actin
MMP-2
ratio of MMP-2/β-actin
MMP-3
ratio of MMP-3/β-actin
MMP-11
ratio of MMP-11/β-actin
MMP-12
ratio of MMP-12/β-actin
MMP-13
ratio of MMP-13/β-actin
MMP-17
ratio of MMP-17/β-actin
MMP-21
ratio of MMP-21/β-actin
N-cadherin
ratio of N-cadherin/β-actin
Bel-7402
Bel-7402-(HUVEC-CM)
MHCC-LM3
MHCC-LM3-(HUVEC-CM)
MHCC-97L
MHCC-97L-(HUVEC-CM)
DU145
DU145-(HUVEC-CM)

Fig. S2

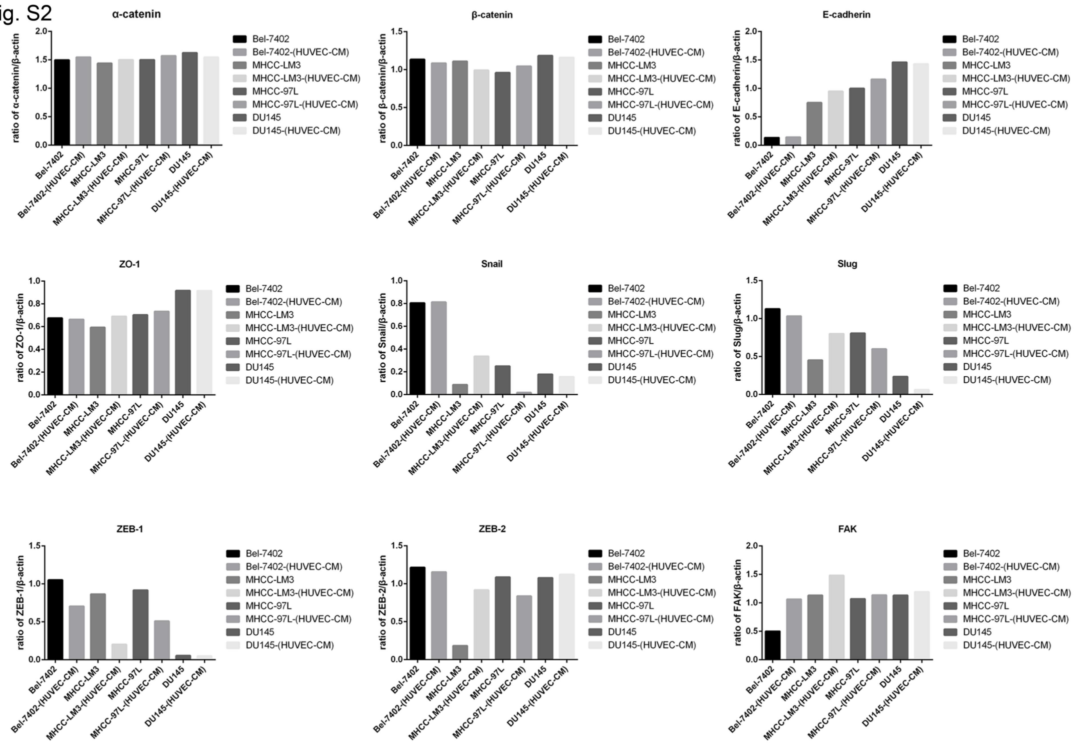
α-catenin
ratio of α-catenin/β-actin
β-catenin
ratio of β-catenin/β-actin
E-cadherin
ratio of E-cadherin/β-actin
ZO-1
ratio of ZO-1/β-actin
Snail
ratio of Snail/β-actin
Slug
ratio of Slug/β-actin
ZEB-1
ratio of ZEB-1/β-actin
ZEB-2
ratio of ZEB-2/β-actin
FAK
ratio of FAK/β-actin
Bel-7402
Bel-7402-(HUVEC-CM)
MHCC-LM3
MHCC-LM3-(HUVEC-CM)
MHCC-97L
MHCC-97L-(HUVEC-CM)
DU145
DU145-(HUVEC-CM)

Fig. S3

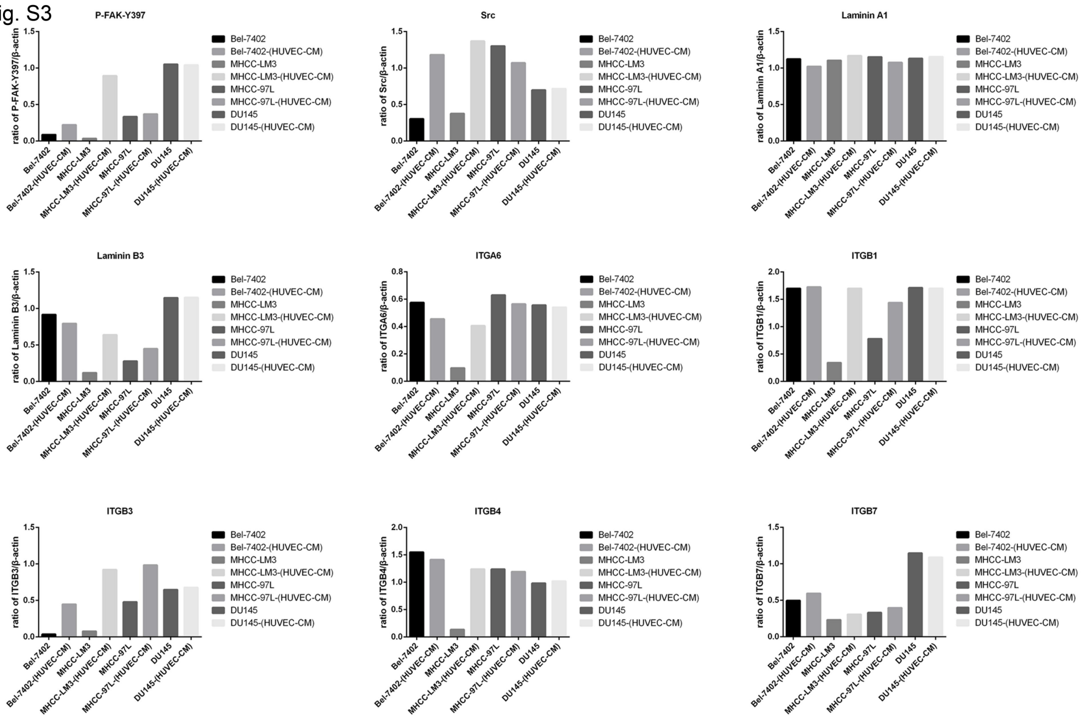
P-FAK-Y397
ratio of P-FAK-Y397/β-actin
Src
ratio of Src/β-actin
Laminin A1
ratio of Laminin A1/β-actin
Laminin B3
ratio of Laminin B3/β-actin
ITGA6
ratio of ITGA6/β-actin
ITGB1
ratio of ITGB1/β-actin
ITGB3
ratio of ITGB3/β-actin
ITGB4
ratio of ITGB4/β-actin
ITGB7
ratio of ITGB7/β-actin
Bel-7402
Bel-7402-(HUVEC-CM)
MHCC-LM3
MHCC-LM3-(HUVEC-CM)
MHCC-97L
MHCC-97L-(HUVEC-CM)
DU145
DU145-(HUVEC-CM)

Fig. S4

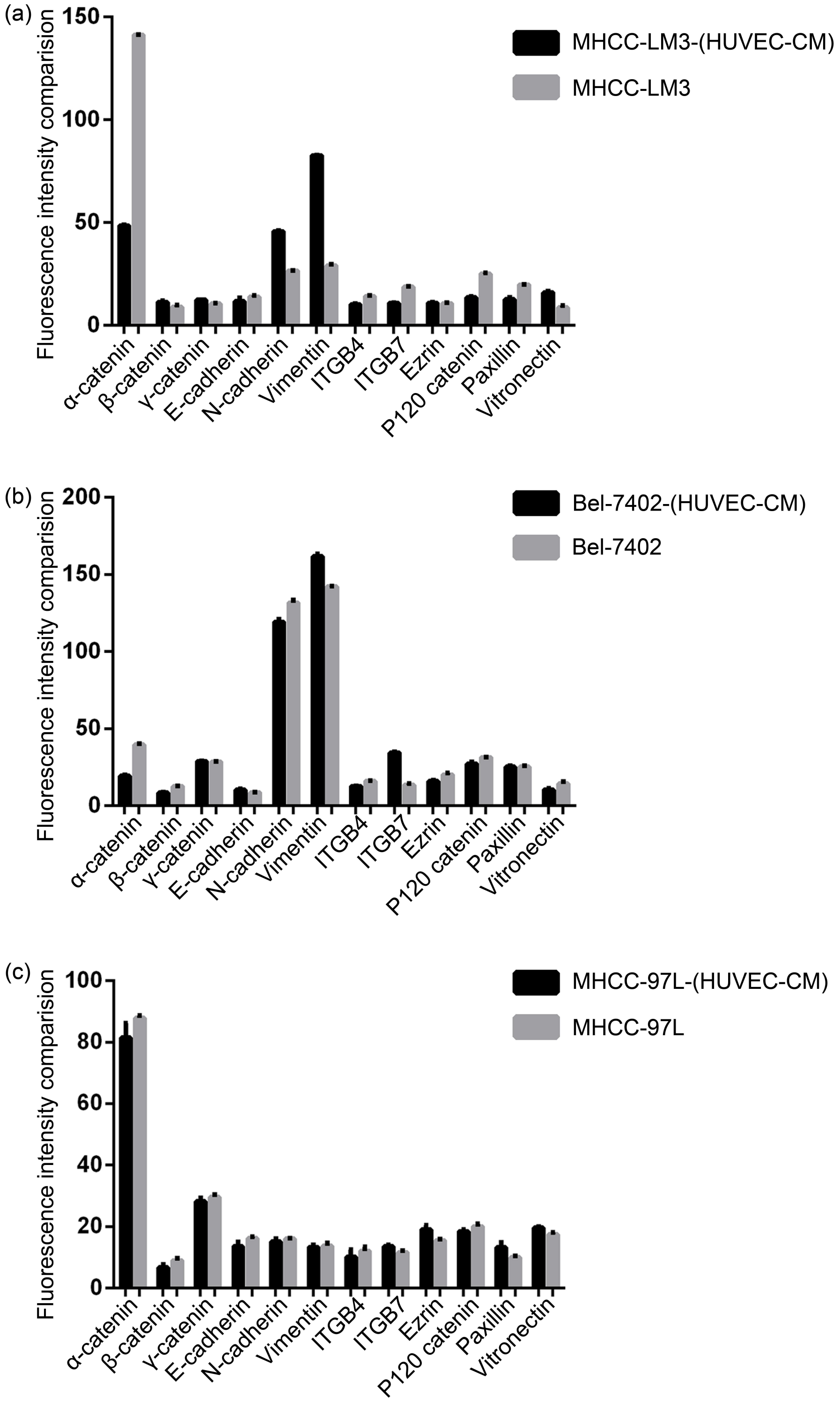
(a)
Fluorescence intensity comparision
150
100
50
0
MHCC-LM3-(HUVEC-CM)
MHCC-LM3
α-catenin
β-catenin
γ-catenin
E-cadherin
N-cadherin
Vimentin
ITGB4
ITGB7
Ezrin
P120 catenin
Paxillin
Vitronectin
(b)
Fluorescence intensity comparision
200
150
100
50
0
Bel-7402-(HUVEC-CM)
Bel-7402
α-catenin
β-catenin
γ-catenin
E-cadherin
N-cadherin
Vimentin
ITGB4
ITGB7
Ezrin
P120 catenin
Paxillin
Vitronectin
(c)
Fluorescence intensity comparision
100
80
60
40
20
0
MHCC-97L-(HUVEC-CM)
MHCC-97L
α-catenin
β-catenin
γ-catenin
E-cadherin
N-cadherin
Vimentin
ITGB4
ITGB7
Ezrin
P120 catenin
Paxillin
Vitronectin

Fig. S5

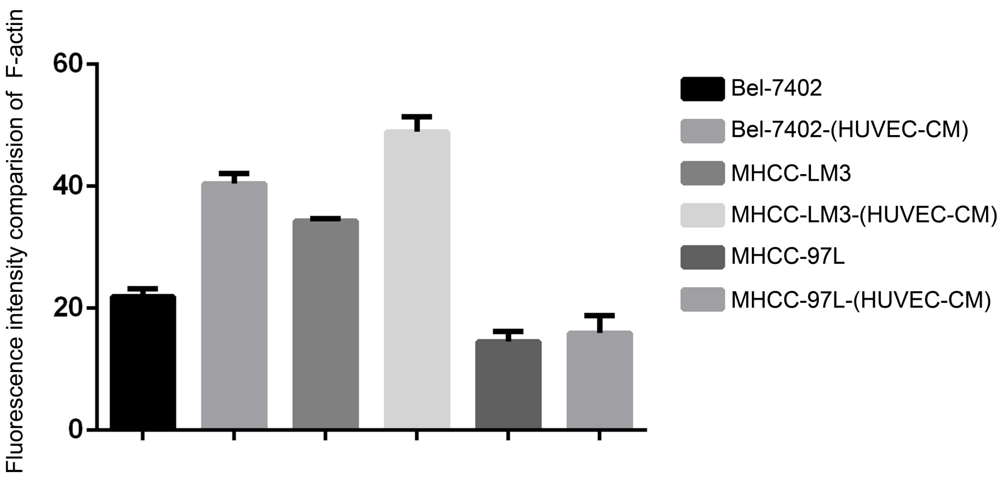
Fluorescence intensity comparision of F-actin
60
40
20
0
Bel-7402
Bel-7402-(HUVEC-CM)
MHCC-LM3
MHCC-LM3-(HUVEC-CM)
MHCC-97L
MHCC-97L-(HUVEC-CM)

Fig. S6

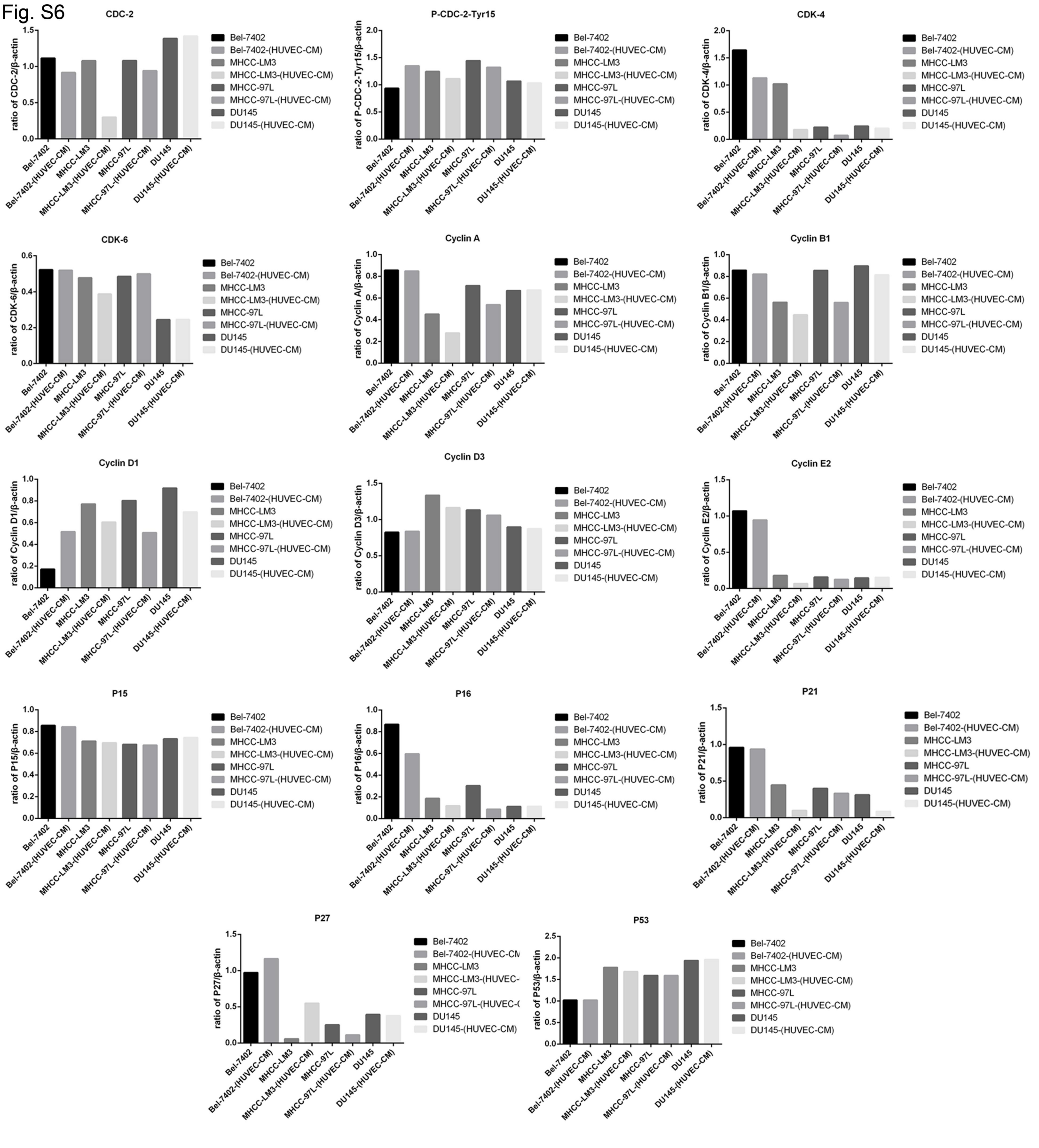
CDC-2
ratio of CDC-2/β-actin
P-CDC-2-Tyr15
ratio of P-CDC-2-Tyr15/β-actin
CDK-4
ratio of CDK-4/β-actin
CDK-6
ratio of CDK-6/β-actin
Cyclin A
ratio of Cyclin A/β-actin
Cyclin B1
ratio of Cyclin B1/β-actin
Cyclin D1
ratio of Cyclin D1/β-actin
Cyclin D3
ratio of Cyclin D3/β-actin
Cyclin E2
ratio of Cyclin E2/β-actin
P15
ratio of P15/β-actin
P16
ratio of P16/β-actin
P21
ratio of P21/β-actin
P27
ratio of P27/β-actin
P53
ratio of P53/β-actin
Bel-7402
Bel-7402-(HUVEC-CM)
MHCC-LM3
MHCC-LM3-(HUVEC-CM)
MHCC-97L
MHCC-97L-(HUVEC-CM)
DU145
DU145-(HUVEC-CM)

Fig. S7

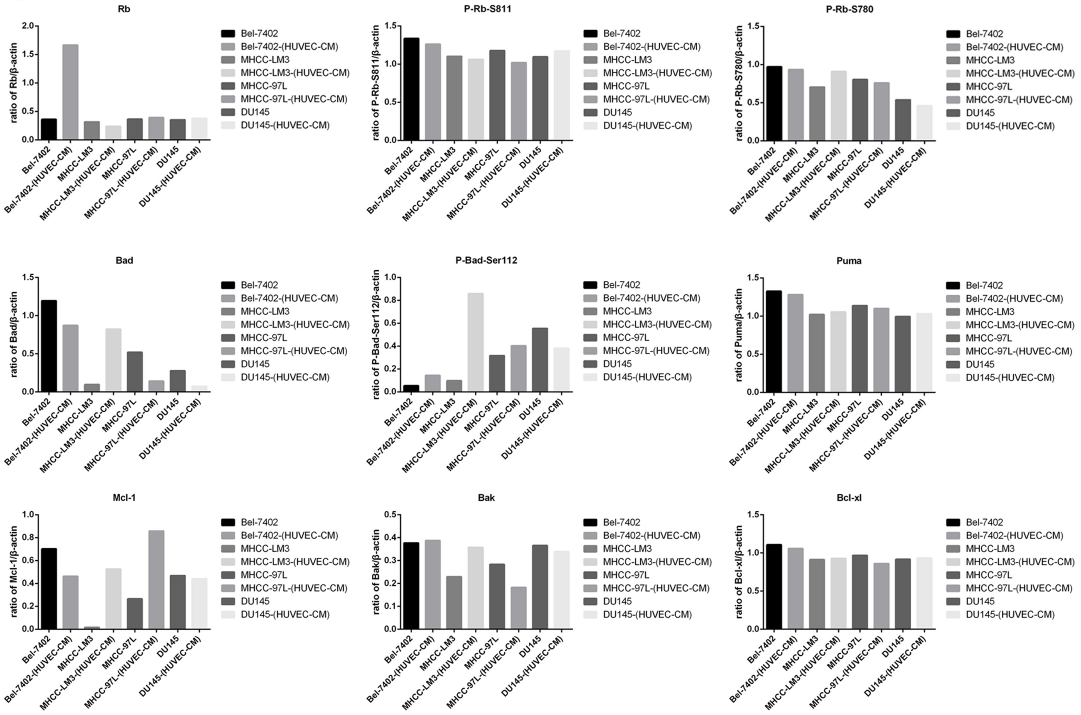
Rb
ratio of Rb/β-actin
P-Rb-S811
ratio of P-Rb-S811/β-actin
P-Rb-S780
ratio of P-Rb-S780/β-actin
Bad
ratio of Bad/β-actin
P-Bad-Ser112
ratio of P-Bad-Ser112/β-actin
Puma
ratio of Puma/β-actin
Mcl-1
ratio of Mcl-1/β-actin
Bak
ratio of Bak/β-actin
Bcl-xl
ratio of Bcl-xl/β-actin
Bel-7402
Bel-7402-(HUVEC-CM)
MHCC-LM3
MHCC-LM3-(HUVEC-CM)
MHCC-97L
MHCC-97L-(HUVEC-CM)
DU145
DU145-(HUVEC-CM)

Fig. S8

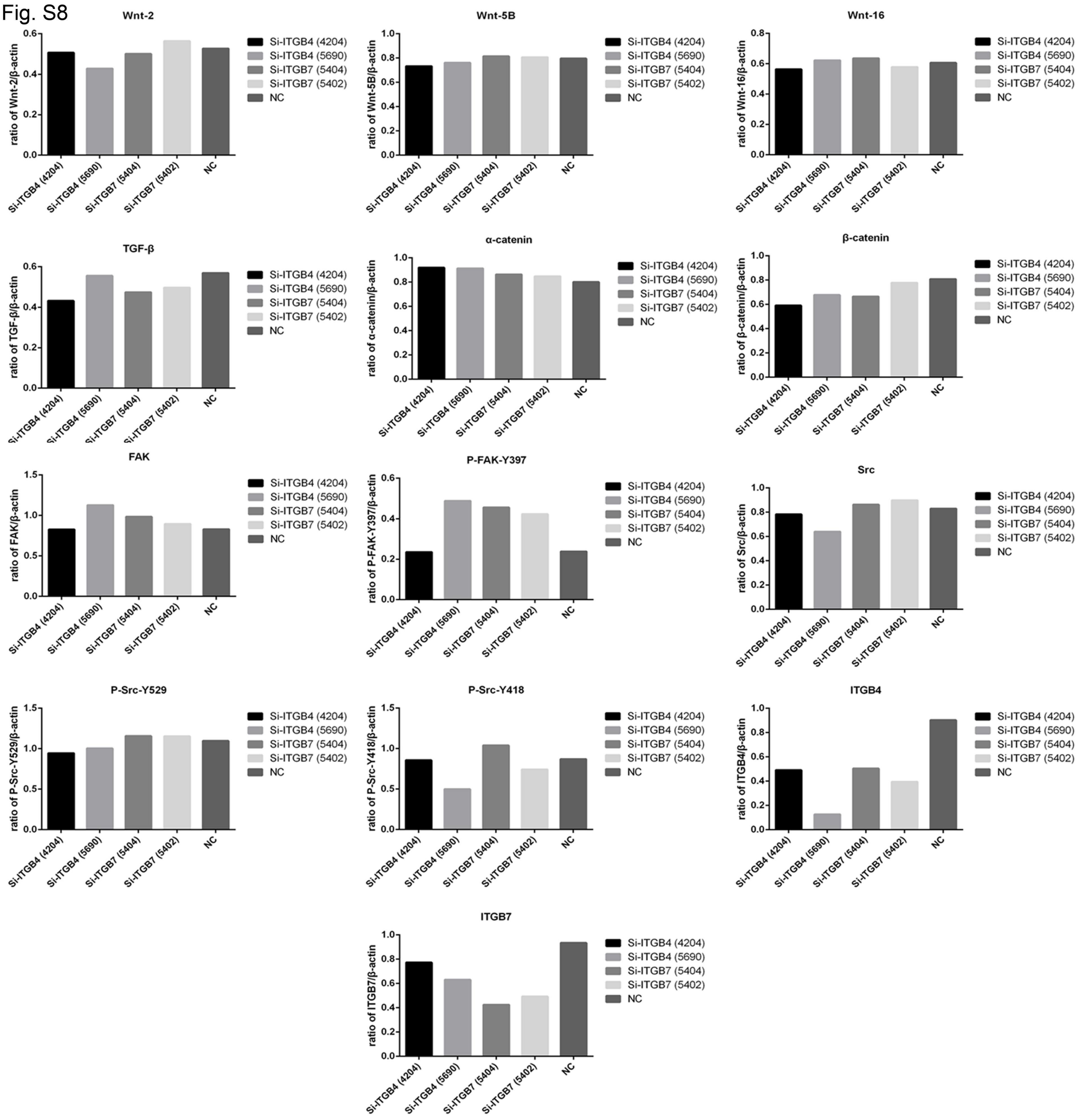

Wnt-2
ratio of Wnt-2/β-actin
Wnt-5B
ratio of Wnt-5B/β-actin
Wnt-16
ratio of Wnt-16/β-actin
TGF-β
ratio of TGF-β/β-actin
α-catenin
ratio of α-catenin/β-actin
β-catenin
ratio of β-catenin/β-actin
FAK
ratio of FAK/β-actin
P-FAK-Y397
ratio of P-FAK-Y397/β-actin
Src
ratio of Src/β-actin
P-Src-Y529
ratio of P-Src-Y529/β-actin
P-Src-Y418
ratio of P-Src-Y418/β-actin
ITGB4
ratio of ITGB4/β-actin
ITGB7
ratio of ITGB7/β-actin
Si-ITGB4 (4204)
Si-ITGB4 (5690)
Si-ITGB7 (5404)
Si-ITGB7 (5402)
NC

Fig. S9

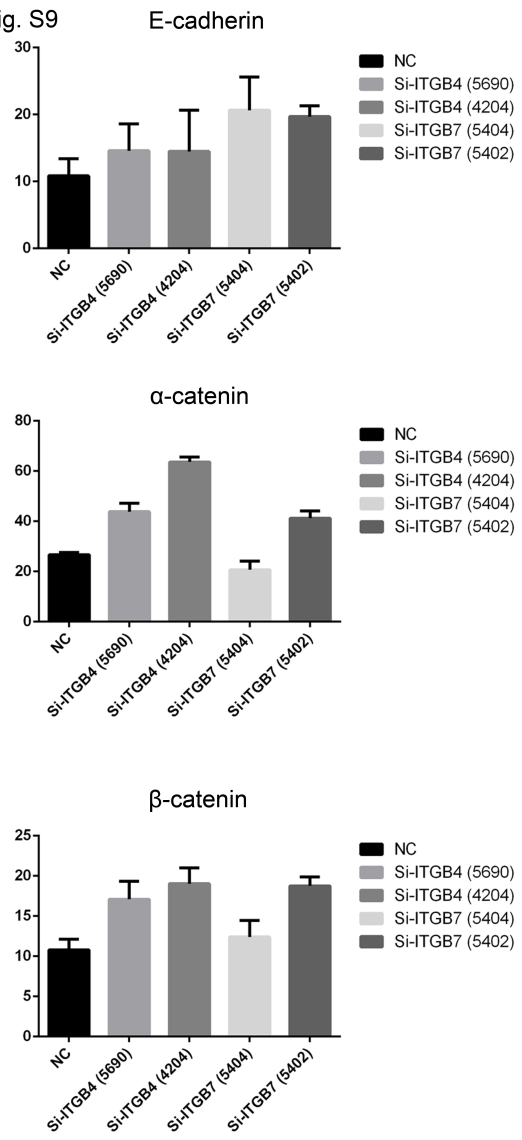
E-cadherin
30
20
10
0
NC
Si-ITGB4 (5690)
Si-ITGB4 (4204)
Si-ITGB7 (5404)
Si-ITGB7 (5402)
α-catenin
80
60
40
20
0
NC
Si-ITGB4 (5690)
Si-ITGB4 (4204)
Si-ITGB7 (5404)
Si-ITGB7 (5402)
β-catenin
25
20
15
10
5
0
NC
Si-ITGB4 (5690)
Si-ITGB4 (4204)
Si-ITGB7 (5404)
Si-ITGB7 (5402)
